# Adherence to Mediterranean Diet and Risk of Type 2 Diabetes: An Updated Systematic Review and Dose–Response Meta-analysis

**DOI:** 10.1016/j.advnut.2025.100562

**Published:** 2025-11-13

**Authors:** Sabina Wallerer, Julia Stadelmaier, Eike Floegel, Eva Kiesswetter, Gina Bantle, Georg Hoffmann, Lukas Schwingshackl

**Affiliations:** 1 Institute for Evidence in Medicine, Medical Center – University of Freiburg/Medical Faculty – University of Freiburg, Freiburg, Germany; 2 Department of Nutritional Sciences, University of Vienna, Vienna, Austria

**Keywords:** Mediterranean diet, adherence, diabetes mellitus, systematic review, meta-analysis

## Abstract

**Background:**

Given the global rise in type 2 diabetes mellitus (T2D), the Mediterranean diet (MedDiet) has gained attention as a promising preventive dietary pattern.

**Objectives:**

This study aims to update and extend our previous systematic review by synthesizing current evidence from randomized controlled trials (RCTs) and prospective cohort studies on the association between MedDiet adherence and incident T2D in adults, and to evaluate the certainty of evidence.

**Methods:**

We conducted a systematic search in MEDLINE, Cochrane CENTRAL, and Scopus from 2014 to May 2025. Eligible studies were prospective cohorts reporting on the adherence to an a priori-defined MedDiet or, for RCTs, MedDiet intervention compared with any other diet and T2D incidence. Random-effects dose–response meta-analyses were performed to estimate hazard ratios (HRs) for MedDiet adherence score. Risk of bias was assessed using the Cochrane risk of bias tool 2 and the Risk Of Bias In Nonrandomized Studies—of Exposures tool, and the certainty of evidence was rated using the Grading of Recommendations Assessment, Development and Evaluation approach.

**Results:**

A total of 24 prospective cohort studies and 1 RCT were included, comprising 991,878 participants and 68,325 T2D cases and a mean follow-up duration of 12.2 y (range: 3.5– 25 y). Higher MedDiet adherence is likely associated with a reduced risk of T2D [HR_per 2-point increment_: 0.92; 95% confidence interval (CI): 0.90, 0.94; moderate certainty]. The dose–response curve shows a consistent decline in T2D risk with higher adherence to MedDiet. The association remained robust over several subgroup analyses, including age, sex, and MedDiet score. The included RCT confirmed the main findings (HR: 0.75; 95% CI: 0.56, 1.01, low certainty).

**Conclusion:**

This updated systematic review and meta-analysis provides moderate-certainty evidence that greater adherence to the MedDiet is associated with a lower risk of T2D. These findings reinforce current dietary guidelines recommending MedDiet as a sound strategy for T2D prevention.

**Statement of Significance**

The aim of the present work is to update and extend our previous systematic review and meta-analysis from 2015 on the adherence to a Mediterranean diet and diabetes risk. This review incorporates all newly published studies, applies refined meta-analytic and methodological techniques, and rates the certainty of evidence using the Grading of Recommendations Assessment, Development and Evaluation approach.

## Introduction

Type 2 diabetes mellitus (T2D) represents a growing global health burden, with both incidence and prevalence expected to rise substantially over the coming decades. According to estimates by the International Diabetes Federation, the number of adults living with diabetes is expected to increase from 589 million in 2024 to 853 million by 2050—an increase of nearly 50% [1]. This trend underscores the urgent need for effective, evidence-based preventive strategies, particularly those targeting modifiable lifestyle factors such as diet.

The Mediterranean diet (MedDiet), originally described by Ancel Keys in the context of the Seven Countries Study, has been extensively investigated for its potential in preventing chronic diseases [2,3]. Its characteristic components include a high consumption of MUFAs (predominantly from extra virgin olive oil), fruits, vegetables, (whole grain) cereals, legumes, and nuts; a moderate intake of fish and alcohol (mainly red wine); and a low intake of red and processed meats [4]. A large body of evidence shows that adherence to the MedDiet is associated with a reduced risk of cardiovascular disease, cancer, neurodegenerative disorders, and all-cause mortality [3,5]. Although moderate alcohol consumption (mainly red wine) has traditionally been considered a component of the MedDiet, data from epidemiological studies suggest that even low levels of alcohol intake are associated with higher risks of certain cancers [6,7]. Accordingly, the health benefits of the MedDiet are attributed to its overall nutrient-rich, plant-based composition rather than alcohol consumption.

In the context of T2D, both randomized controlled trials (RCTs) and observational studies have consistently reported beneficial effects of MedDiet on glycemic control, insulin sensitivity, and diabetes incidence [8,9]. A previous systematic review and meta-analysis conducted in 2015 by our group synthesized the available evidence from RCTs and prospective cohort studies, concluding that high adherence to the MedDiet was inversely associated with T2D risk [10].

Since the publication of our 2015 review, a substantial number of additional studies have been published, allowing for a more comprehensive and statistically robust synthesis of the evidence. In the present work, we applied updated methodological techniques to assess risk of bias and rate certainty of evidence, and used comprehensive analytical approaches to reassess the relationship between MedDiet adherence and T2D risk. These refinements enhance the methodological rigor and interpretability of our findings, and contribute to a more reliable evidence base for dietary recommendations in public health and clinical practice.

Therefore, the aim of the present work is to update and extend our previous systematic review and meta-analysis by incorporating newly published studies, applying refined meta-analytic and methodological techniques, and rating the certainty of the association between adherence to a MedDiet and risk of T2D in adults.

## Methods

We report this systematic review according to the PRISMA guideline [11] and the PRISMA Statement for Reporting Literature Searches in Systematic Reviews [12]. The methodological approach was predefined based on the framework of our previous systematic review [10], and further informed by state-of-the-art approaches for evidence syntheses.

### Data sources and searches

We conducted a comprehensive systematic literature search in 3 electronic databases, including MEDLINE (via OVID), Cochrane CENTRAL, and Scopus, from 2014 to 26 May, 2025. No language restrictions were applied. The detailed search strategies can be found in Supplemental Appendix 1.

Additionally, we conducted backward citation tracking on systematic and narrative reviews, identified through our searches, as well as on all included studies.

## Eligibility criteria

We included studies fulfilling the following eligibility criteria:

Population: adults (aged ≥18 y); generally healthy. We excluded study populations with a particular condition, such as prediabetes, metabolic dysfunction–associated steatotic liver disease, chronic kidney disease, or cancer, to ensure a more homogeneous study population and thereby enhance the generalizability of the results. We excluded studies involving exclusively infants, children, adolescents, or pregnant women.

Intervention/exposure and comparison: trials investigating a MedDiet intervention compared with any other diet or prospective observational studies evaluating the association of an a priori score used for assessing adherence to a MedDiet [e.g., traditional MedDiet (tMED) [13] or the alternate MedDiet score (aMED) [14]].

Outcome: T2D incidence.

Study design: we considered RCTs and prospective observational studies (e.g., cohort, case-cohort, nested case-control).

Detailed eligibility criteria are displayed in Supplemental Table 1.

### Study selection

After deduplication of search hits using Systematic Review Accelerator [15], 2 reviewers from a group of 5 (EK, GB, JS, LS, SW) independently screened each title/abstract and full text for potentially eligible publications. On the full text level, reasons for exclusion were recorded. The screening process was implemented using the Covidence systematic review software [16]. Any disagreements were resolved by discussion or with the help of a third reviewer (LS) if no agreement could be reached. If multiple publications investigated the same RCT or cohort, the one with a larger number of T2D cases followed by the one with longer follow-up was included. Conference abstracts with adequate information on methods and results were also considered eligible.

### Data extraction

After identification of eligible publications, 2 reviewers (LS, SW) extracted the data independently in a piloted data extraction form (Microsoft Excel). Conflicts were solved by discussion with a third reviewer (EK or JS) if no agreement could be reached. We extracted the following data: first author's name, RCT or cohort name, year of publication, location, study design, age, sex, BMI (in kg/m^2^), description of population, number of participants, length of follow-up, outcome assessment, number of cases, exposure assessment (type and number of assessments (i.e., at baseline or repeated)), description of intervention (for RCTs), definition of MedDiet including score range and components, covariate adjustment set, risk estimate with 95% confidence interval (95% CI). If a study presents several risk estimates, the one with maximal adjustment was chosen. However, estimates that included adjustment for BMI were not prioritized, as BMI is considered a potential mediator in the causal pathway between MedDiet adherence and T2D risk, and adjusting for it could lead to overadjustment bias [17,18].

If studies reported the relevant data only in figures, we used the Web plot digitizer [19] for extraction.

### Risk of bias assessment

Two reviewers from a group of 4 (EF, GB, JS, SW) independently assessed risk of bias (RoB) of each included study, and any disagreements were resolved by consensus. For RCTs, we used the Cochrane risk of bias tool (RoB 2) [20], and for prospective observational studies, the Risk Of Bias In Nonrandomized Studies—of Exposures tool (ROBINS-E) [18]. RoB assessments were visualized using the robvis tool [21].

The RoB 2 assessment includes 5 domains: *1*) randomization process; *2*) deviations from intended interventions; *3*) missing outcome data; *4*) measurement of the outcome; *5*) selection of the reported results.

ROBINS-E assessment includes 7 domains of bias: *1*) confounding, *2*) measurement of the exposure, *3*) selection of participants into the study (or into the analysis), *4*) postexposure interventions, *5*) missing data, *6*) measurement of the outcome, and *7*) selection of the reported results. We used a triage approach [22] if a study did not adjust for all prespecified basic confounders (age, sex, smoking, education/socioeconomic status, energy intake, and physical activity). In this case, other domains were not assessed because the overall judgment was not influenced any further [18]. We judged each domain as well as the overall RoB as low, some concerns, and high or very high RoB. Details of the RoB 2 and ROBINS-E assessment are provided in Supplemental Appendix 2 and 3.

### Statistical analysis

The present update focused on dose–response meta-analyses instead of high compared with low comparisons to capture the shape and strength of the association of MedDiet adherence and T2D.

If >1 RCT reported effect estimates for MedDiet compared with control, a random-effects meta-analysis was conducted to estimate the effect of MedDiet on T2D risk. Cohort studies that reported hazard ratios (HRs) directly for a 2-point increment for the standard 9-point scale were included as such. For studies reporting HRs per any other point increment, we converted the estimates by calculating the logHR and 95% CI/1 point increment, multiplying both by 2, and exponentiating the results. If alternative scoring ranges/systems (e.g., 10- or 18-point scales) were used in the original publications, we applied linear scaling to approximate a 2-point increment on the 9-point scale (e.g., 2 points in an 18-point scale were treated as 1 point on the 9-point scale). For studies reporting categorical adherence data only, we used the method proposed by Greenland et al. [23] to estimate the continuous association for a 2-point increment.

We then conducted a random-effects dose–response meta-analysis to estimate the pooled HR for each 2-point increment in MedDiet adherence score.

Using all studies that reported categorical adherence data, we performed a 1-stage mixed-effects meta-analysis to characterize the shape of the dose–response relationship between MedDiet adherence and T2D risk [24]. To enable comparability, we converted all exposure categories to the 9-point scale, using published or estimated median values within adherence categories. For example, scores of 3.5, 9, and 14.5 on an 18-point scale were rescaled to their corresponding equivalents on a 9-point scale. We performed dose–response analysis using restricted cubic splines with 3 knots at the 10th, 50th, and 90th percentiles. We tested linearity assumption using the likelihood ratio test and the Wald test [24].

Between-study heterogeneity was assessed using the Cochrane Q test and quantified with the I^2^ statistic [25]. Additionally, 95% prediction intervals were calculated to estimate the expected range of true effects in future studies [26].

We used funnel plots and Egger’s linear regression test for funnel plot asymmetry to evaluate dissemination bias and small study effects [27].

Prespecified subgroup analyses (if ≥10 studies were available) were performed for: age, sex, geographical location, follow-up, number of cases, MedDiet score, and covariate adjustment [e.g., BMI (yes/no), smoking (yes/no)]. To examine the robustness of our findings, a sensitivity analysis was conducted by excluding studies with high RoB.

### Certainty of evidence

The certainty of evidence was evaluated using the Grading of Recommendations Assessment, Development and Evaluation (GRADE) approach. The GRADE approach considers RoB, inconsistency, indirectness, imprecision, and publication bias [28]. For observational studies, in addition to the magnitude of the effect and the presence of a dose–response relationship is taken into account [29].

We rated the certainty of evidence separately for RCTs and prospective observational studies. Two reviewers (LS, SW) independently assessed the certainty of evidence, and any discrepancies were resolved through consensus.

## Results

The database searches resulted in 3257 references. After deduplication, we screened the eligibility of 2217 titles/abstracts and in a subsequent step, 46 full-texts. Reasons for exclusion of full-texts are given in Supplemental Tables 2 and 3. One study [30] was excluded because of serious concerns about data integrity. Notably, 4 of 9 studies that were included in our previous systematic review [10] were excluded from the present update: Tobias et al. [31] due to inclusion of women with a history of gestational diabetes; Mozaffarian et al. [32] because it included participants with prior myocardial infarction; Brunner et al. [33] due to use of principal component analysis rather than an a priori MedDiet score; and Martínez-González et al. [34] due to overlap of study participants (Supplemental Table 3).

**FIGURE 1:**
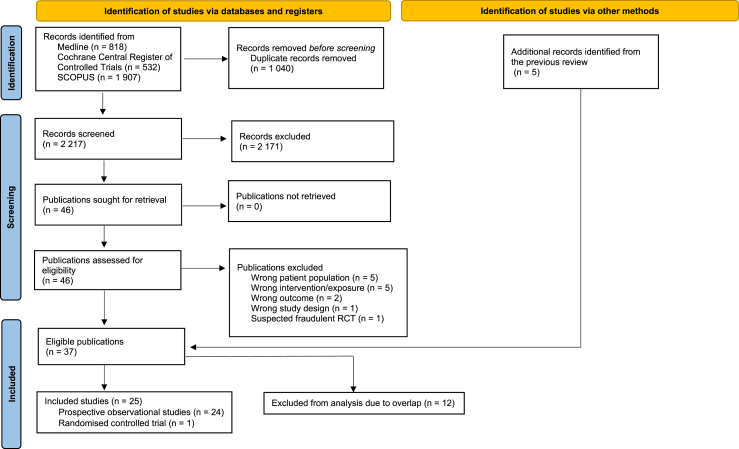
PRISMA 2020 flow chart of the process for study selection. RCT, randomized controlled trial.

The flow diagram of the search and screening process is depicted in Figure 1. Finally, we included 24 prospective observational studies [[35], [36], [37], [38], [39], [40], [41], [42], [43], [44], [45], [46], [47], [48], [49], [50], [51], [52], [53], [54], [55], [56], [57], [58]] with 988,337 participants and 68,052 T2D incidence cases, and 1 RCT [59,60] with 3541 participants and 273 T2D incidence cases.

### Study characteristics

Detailed information on the study characteristics can be found in Supplemental Tables 4 and 5. The included RCT regards the PREDIMED trial. This trial was conducted in Spain and compared either a MedDiet enriched with extra virgin olive oil or nuts compared with a low-fat diet over 4.1 y [59,60].

Nine cohort studies were conducted in the United States [[35], [36], [37],^39^,^44^,^46^,^47^,^50^,^54^], 8 in Europe [40,42,43,48,52,53,56,57], 5 in Asia [38,41,49,51,55], and 2 in Australia [45,58]. In all cohort studies except 2 (using consecutive 24-h recalls or dietary records) [42,55], diet was assessed using validated food-frequency questionnaires or diet history questionnaires. In 5 cohort studies [39,42,48,50,55], diet was assessed at multiple time points, and averages (or measures) of intake were used for the analysis. One cohort study used repeated dietary measurements for some, but not all, participants [44]. The mean follow-up duration was 12.2 y (range, 3.5–25), and most included both men and women.

Ten cohort studies used the tMED score as exposure [40,41,43,45,[53], [54], [55], [56], [57], [58]], another 10 used the aMED score [35,36,38,39,42,44,46,47,49,50], and 5 studies used adapted MedDiet scores (e.g., South Asian MedDiet score, Americanized MedDiet score) [37,48,51,52,60].

The PREDIMED trial was rated with some concerns for the overall RoB. Concerns regarding the RoB were raised due to potential bias arising from the randomization process, due to deviations from intervention, and missing outcome data (Supplemental Figure 1). According to our evaluation, 15 cohort studies had some concerns in the overall RoB [[35], [36], [37], [38],[42], [43], [44],^46^,^47^,[50], [51], [52],^54^,^55^,^57^], and 9 were judged as high RoB [[39], [40], [41],^45^,^48^,^49^,^53^,^56^,^58^]. The reason for high RoB was insufficient adjustment of confounders in all 9 cohort studies (Supplemental Figure 2).

### Mediterranean dietary pattern and T2D

#### Randomized controlled trial evidence

**FIGURE 2:**
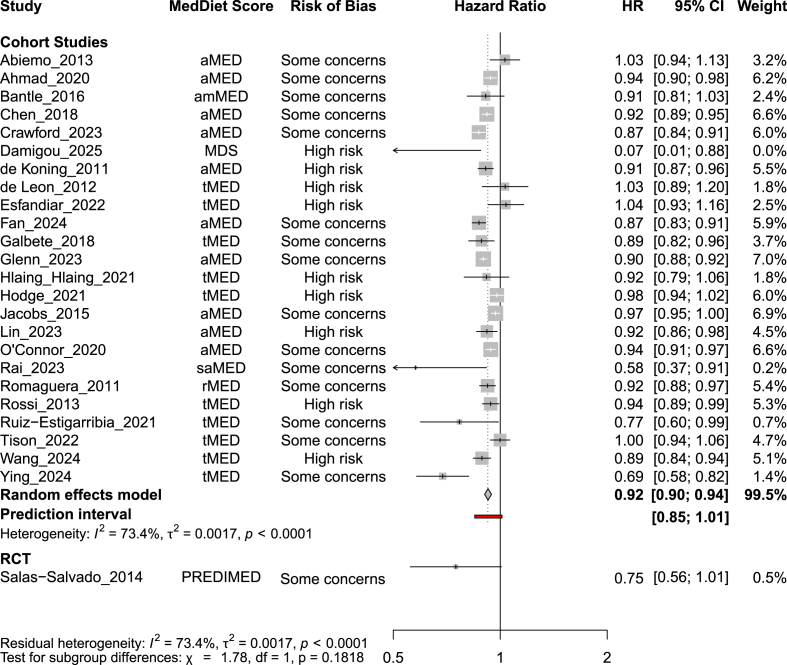
Forest plot showing the HR and 95% CI of T2D per 2-unit increment of Mediterranean diet score in 24 prospective cohort studies (988,337 participants), and comparing a Mediterranean dietary intervention (vs. low-fat diet) for 1 randomized controlled trial (RCT, 3541 participants). For each study, the gray square indicates the HR, the size of which indicates the study’s weight in the analysis (weights are from random-effects analysis using inverse variance weighing) and the horizontal line represents the 95% CI. The center of the diamond indicates the summary estimate of the HR and its width represents the 95% CI of the summary HR estimate. CI, confidence interval; aMED, alternate Mediterranean diet score; amMED, Americanized Mediterranean diet score; HR, hazard ratio; MDS, Mediterranean diet score; PREDIMED, Prevención con Dieta Mediterránea; T2D, type 2 diabetes mellitus; tMED, traditional Mediterranean diet score; saMED, South Asian Mediterranean diet score; RCT, randomized controlled trial.

One RCT was eligible to be included into the present review. On the basis of low certainty evidence, MedDiet may reduce risk of T2D compared with a low-fat diet (HR: 0.75; 95% CI: 0.56, 1.01; Figure 2, Table 1).

**TABLE 1:** **GRADE assessment for Mediterranean diet adherence and type 2 diabetes mellitus risk** for included randomized controlled trials and prospective observational studies

Certainty assessment							No of patients		Effect		Certainty
No of studies	Study design	Risk of bias	Inconsistency	Indirectness	Imprecision	Other considerations	High adherence	Low adherence to MedDiet	Relative (95% CI)	Absolute (95% CI)	
T2D incidence (RCTs)											
1	Randomized trials	Serious^1^	Not serious	Not serious	Serious^2^	None	149/2077 (7.2%)	80/906 (8.8%)	HR 0.75 (0.56, 1.01)	21 fewer per 1.000 (from 38 fewer to 1 more)	⊕⊕⊕◯◯ Low^1^^,^^2^
T2D incidence (prospective observational studies)											
24	Nonrandomized studies	Serious^3^	Not serious	Not serious	Not serious^4^	None	—	68,052/988,337 (6.9%)	HR 0.92 (0.90, 0.94)	5 fewer per 1.000 (from 7 fewer to 4 fewer)	⊕⊕⊕◯ Moderate^3^
									Comparing 6-point vs. 1-point adherence HR 0.86 (0.83, 0.90)	9 fewer per 1000 (from 11 fewer to 7 fewer)	
									Comparing 8-point vs. 1-point adherence HR 0.79 (0.73, 0.85)	14 fewer per 1000 (from 18 fewer to 10 fewer)	

Question: High adherence compared with low adherence to MedDiet for diabetes prevention.

Abbreviations: CI, confidence interval; GRADE, Grading of Recommendations Assessment, Development and Evaluation; HR, hazard ratio; MedDiet, Mediterranean diet; RCT, randomized controlled trial; RoB, risk of bias; T2D, type 2 diabetes mellitus.

1 Downgraded by 1 level for RoB: the study was judged with some concerns for RoB.

2 Downgraded by 1 level because 95% CI overlaps the null effect. The minimal important difference is defined as 5 fewer or more/per 1000.

3 Downgraded by 1 level for RoB: <2/3 of the studies (and their contributing weight) were rated with a low RoB, and < 2/3 of the studies (and their contributing weight) were rated with a high RoB. Specified for the present comparison: 9/24 studies with high RoB (32.7% sum of weight), sensitivity analysis excluding studies with high RoB shows a robust estimate.

4 Not downgraded for imprecision: although the 95% CI of the absolute effect slightly overlaps the minimal important difference, the effect comparing 6- or 8-point adherence to the Mediterranean diet vs. 1 point adherence was substantially larger and the corresponding 95% CI did not overlap of the minimal important difference.

#### Observational evidence

**FIGURE 3:**
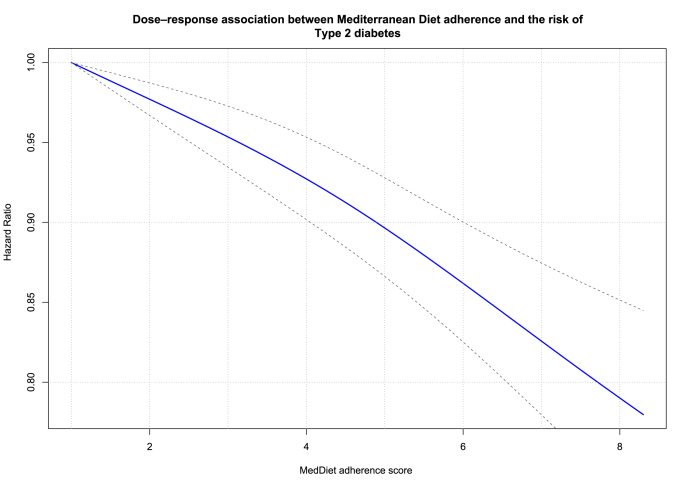
Dose–response association between Mediterranean diet adherence and risk of type 2 diabetes mellitus (*n* = 22 prospective observational studies). The blue line represents the dose–response relationship (established using restricted using splines with knots at the 10th 50th, and 90th percentiles), and dashed lines represent 95% confidence interval. *P* values for nonlinearity tests: *P* value_likelihood ratio test_ = 0.02, *P* value_Wald type test_ = 0.26. MedDiet, Mediterranean diet.

Each 2-point increment in the MedDiet adherence score was likely associated with a decrease in T2D risk by 8% (HR: 0.92; 95% CI: 0.90, 0.94; moderate certainty of evidence, 95% prediction interval: 0.85 to 1.01, Figure 2, Table 1). Twenty-two studies provided categorical data and were therefore eligible to be included in the 1-stage dose–response meta-analysis using restricted cubic splines. The dose–response curve shows a consistent dose–response relationship, with greater reductions for T2D risk at higher adherence score levels (Figure 3). The HRs for MedDiet scores of 2, 4, 6, and 8 were HR 0.98 (95% CI: 0.97, 0.99), HR 0.93 (95% CI: 0.90, 0.95), HR 0.86 (95% CI: 0.83, 0.90), and HR 0.79 (95% CI: 0.73, 0.85), respectively. The certainty of evidence was rated moderate due to downgrading once for serious RoB (Figure 3, Table 1).

### Subgroup and sensitivity analyses

All subgroup analyses indicated a robust association with a lower risk of T2D, with no indication of interaction effects (Table 2). The results were similar for men and women, and no difference was observed when comparing aMED and tMED scores (test for subgroup differences: *P* = 0.63) (Supplemental Figure 3, Table 2). The sensitivity analysis excluding high RoB studies did not alter the main results (HR: 0.92; 95% CI: 0.89, 0.94; Supplemental Figure 4, Table 2). European Prospective Investigation into Cancer and Nutrition (EPIC)-Potsdam [43] and EPIC-Norfolk [56] accounted for 7% and 9% of EPIC-InterAct [52] cases, respectively, indicating a small participant overlap. Therefore, we conducted an explorative sensitivity analysis excluding EPIC-Potsdam and EPIC-Norfolk to test the robustness of our results (Supplemental Figure 5). This analysis did not alter the main results. No indication for publication bias was observed by Egger’s linear regression test (*P* = 0.55) or inspection of the funnel plot (Supplemental Figure 6).

**TABLE 2:** Subgroup analyses for Mediterranean diet adherence and type 2 diabetes mellitus risk

	Number of studies	Hazard ratio (95% CI)	Heterogeneity I^2^ (%)	Test for interaction (*P* value)
Age				
>55	10	0.93 (0.90, 0.96)	84.7	*P* = 0.82
≤55	14	0.92 (0.89, 0.95)	52.3	
Sex				
Men	8	0.94 (0.91, 0.97)	50.1	*P* = 0.47
Women	11	0.92 (0.90, 0.95)	74.4	
Location				
United States	9	0.94 (0.91, 0.97)	80.2	*P* = 0.40
Asia	5	0.90 (0.85, 0.96)	79.3	
Europe	8	0.90 (0.87, 0.94)	51.6	
Australia	2	0.96 (0.89, 1.04)	0	
Years of follow-up				
<10	8	0.95 (0.91, 0.99)	75.9	*P* = 0.21
≥10	16	0.92 (0.89, 0.94)	66.0	
Number of cases				
<2000	16	0.92 (0.89, 0.95)	75.2	*P* = 0.81
≥2000	8	0.93 (0.90, 0.96)	68.1	
Mediterranean diet score				
aMED	10	0.92 (0.89, 0.95)	78.6	*P* = 0.63
tMED	10	0.93 (0.90, 0.97)	72.7	
Adjustment factor (yes/no)				
BMI				
Yes	14	0.91 (0.88, 0.94)	69.7	*P* = 0.36
No	10	0.93 (0.90, 0.97)	78.7	
Smoking				
Yes	22	0.92 (0.90, 0.94)	75.0	*P* = 0.33
No	2	0.96 (0.88, 1.05)	26.7	
Alcohol				
Yes	4	0.92 (0.87, 0.98)	74.7	*P* = 0.99
No	20	0.92 (0.90, 0.95)	73.1	
Physical activity				
Yes	23	0.92 (0.90, 0.94)	74.0	*P* = 0.19
No	1	1.03 (0.89, 1.20)	—	
Energy intake				
Yes	18	0.92 (0.90, 0.94)	76.1	*P* = 0.56
No	6	0.94 (0.89, 0.99)	63.5	
Family history of type 2 diabetes mellitus				
Yes	16	0.94 (0.91, 0.96)	70.5	*P* = 0.14
No	8	0.94 (0.89, 0.99)	70.1	

Abbreviations: aMED, alternate Mediterranean diet score; CI, confidence interval; tMED, traditional Mediterranean diet score.

## Discussion

This updated systematic review and meta-analysis included 1 RCT and 24 prospective observational studies, encompassing nearly 1 million participants, to evaluate the association between adherence to the MedDiet and risk of T2D. The RCT evidence was rated as low certainty due to RoB and imprecision, whereas the prospective observational evidence was rated as moderate certainty (downgraded once for RoB). The results indicate an ∼8% lower risk of T2D per a 2-unit increment to MedDiet adherence.

The inverse relationship between MedDiet adherence and T2D risk remained robust in subgroup analyses of studies adjusting for key confounders, including sex and total energy intake. Notably, the association persisted even in studies controlling for family history of T2D, suggesting a potential independent beneficial association of MedDiet adherence. Dose–response analyses of observational studies indicated a progressive decline of T2D risk with higher levels of adherence to the MedDiet, corresponding to a 21% lower risk when comparing an adherence score of 8 points to the lowest adherence category (score of 1). Although RCT and observational evidence were similar regarding population, intervention/exposure, control, outcome characteristics and effect estimates were congruent, we did not pool estimates due to the difference in certainty of evidence.

Overall, the findings of this updated review are in line with those of our previous systematic review published in 2015, including 8 prospective observational studies and 1 RCT [10]. In that analysis, high adherence to the MedDiet was associated with a 19% lower risk of T2D, and each 2-point increase in the tMED score corresponded to a 7% reduction in T2D risk. However, the current update substantially expands the evidence base by incorporating data from 20 additional prospective cohort studies. Compared with the earlier review, the updated analysis now covers a significantly larger population—991,878 compared with 122,810 participants—and a substantially higher number of incident T2D cases (68,325 compared with 19,976).

Importantly, this updated systematic review is more homogeneous with respect to participant characteristics and the operationalization of the MedDiet adherence score, thereby enhancing the comparability across studies. Therefore, 4 studies included in the 2015 review were excluded from the current analysis.

Although our 2015 systematic review was the first to comprehensively evaluate the association between adherence to the MedDiet and risk of T2D, several subsequent reviews have since been published in the scientific literature.

For instance, 1 systematic review based solely on a high compared with low adherence comparison reported an 11% lower risk of T2D [61]. However, this review included only 248,140 participants, did neither perform dose–response analyses, nor assess RoB or rate the certainty of evidence.

Another review by Sarsangi et al. [62] included 16 cohort studies with 759,806 participants and found a 3% decrease in T2D risk/1-point increase in MedDiet adherence score. However, the review was limited by not including all eligible studies, and by assessing study quality using the Newcastle–Ottawa Scale [63] rather than a structured RoB tool [20]. Additionally, no subgroup analyses were reported for the dose–response relationship. Finally, overall, only 11 studies were included in the dose–response meta-analysis, potentially limiting the robustness of the findings.

Similarly, the review by Zeraattalab-Motlagh et al. [64], included 13 prospective cohort studies with 410,303 participants and 41,466 T2D cases, and reported a relative risk of 0.86 (95% CI: 0.82, 0.91) per 2-point increase in MedDiet adherence score. In our updated analysis, which included 24 prospective studies and 1 RCT, we observed a more conservative estimate (HR: 0.92). The discrepancy in effect size is mainly driven by the inclusion of updated reports from the ATTICA epidemiological study, the Tehran Lipid and Glucose Study (TLGS), and UK Biobank cohorts in our review. Notably, although Zeraattalab-Motlagh et al. [64] conducted both a Risk Of Bias In Non-randomized Studies—of Interventions tool-based RoB assessment and rated the certainty of evidence using the GRADE approach (moderate certainty), they did not include any RCTs and restricted subgroup analyses to high compared with low adherence comparisons, without further stratification within the dose–response analysis.

Our findings are also in line with previous evidence on other a priori diet quality indices. A recent systematic review reported consistent inverse associations between higher adherence to the Healthy Eating Index (HEI), the alternate HEI, and the dietary approaches to stop hypertension score and the risk of T2D [65]. The reported inverse associations ranged from 12% to 22%, depending on the specific index and adherence level. These findings support the notion that overall diet quality—as captured by various dietary scoring systems—plays a meaningful role in T2D prevention and further reinforce the protective potential of the MedDiet [65].

Among the studies included in our review, only the study by Ying et al. [55] reported the associations between individual food groups contributing to the MedDiet score and the risk of T2D. In this analysis, higher intakes of fruits, nuts, and fish were each inversely associated with T2D risk. These findings are supported by evidence from an umbrella review by Neuenschwander et al. [66], which showed an inverse association with T2D for several food groups relevant to the MedDiet. Specifically, high intake of whole grains, and olive oil and moderate wine intake was associated with a reduced risk of T2D.

### Mechanism

With regard to possible mechanisms of action of the MedDiet, interactions between endothelial dysfunction, oxidative stress, and inflammation have been reported. Two meta-analyses reported an increased flow-mediated dilatation after a MedDiet when compared with either a control diet [67] and in studies where a high intake of olive oil was compared with omega-3 (n-3) fatty acid supplementation [68]. Chronic low-grade inflammation has been postulated to be a predictor of the development of type 2 diabetes. The phenolic compounds in extra virgin olive oil can antagonize oxidative stress by inhibiting inflammatory mediators [69] and improve the vasodilatory endothelial response by facilitating the availability of NO [70]. An anti-inflammatory effect of polyphenols has also been demonstrated by the downregulation of IL-6, inducible nitric oxide synthase or serum C-reactive protein [71]. In addition to phenolic compounds, MUFAs from olive oil have also been associated with an anti-inflammatory effect, such as inhibition of nuclear factor ‘kappa-light-chain-enhancer’ of activated B-cells (NF-κB) or cyclooxygenases [72].

Obesity (especially high amounts of visceral fat) exerts detrimental effects of insulin sensitivity via the production and release of proinflammatory adipocytokines. Therefore, a long-term effect of the MedDiet on weight management/weight loss might represent another benefit in the prevention of type 2 diabetes [73]. In a recent study by Ruiz-Canela et al. [74], reducing type 2 diabetes incidence by a MedDiet could be further improved by combining the diet with caloric restriction and physical activity.

A typical MedDiet is rich in fiber used in microbiota to produce short-chain fatty acids, which have been shown to prevent weight gain and to improve insulin sensitivity, albeit mostly in studies with manifested type 2 diabetes [[75], [76], [77], [78]].

Another mechanism of action could be that certain food groups are used only sparingly in the daily diet. In the case of the MedDiet, this would include red and processed meat. A positive association between the consumption of red and processed meat and the onset of T2D was found in an umbrella review by Banjarnahor et al. [79]. The authors refer to poor protein quality and higher consumption of SFAs as potential explanations.

### Strengths and limitations

This updated systematic review has several strengths. It includes the largest number of prospective cohort studies to date—along with 1 RCT—covering nearly 1 million participants and 68,000 T2D cases. By focusing on dose–response relationships rather than high-versus-low comparisons, we provide a more nuanced and comparable risk estimate across studies. We applied robust methodological tools, including ROBINS-E, RoB 2, and the GRADE approach, to assess RoB and rate the certainty of evidence. Importantly, we excluded studies in diseased populations or those using data-driven dietary patterns to enhance internal validity. The association remained robust in subgroup analyses controlling for key confounders.

Despite its strengths, this review also has several limitations. First, as in all evidence syntheses of observational studies, residual confounding cannot be fully ruled out. Although most included cohorts adjusted for key confounders such as BMI, physical activity, smoking, and energy intake, the completeness and quality of confounder control varied, and not all studies accounted for family history of T2D. However, the inclusion of 1 RCT—which demonstrated consistent findings with observational evidence—strengthens the certainty and lends support to a potential causal relationship. Second, the operationalization of the MedDiet differed across studies in terms of scoring systems, cut-off values, and included food groups, introducing potential heterogeneity in exposure measurement despite our efforts to harmonize scores to a common 9-point scale. However, the subgroup analysis in terms of types of MedDiet scores confirmed the findings of the main analysis. Third, although we excluded studies in diseased populations and those based on data-driven patterns, there remains a risk of misclassification of dietary intake due to reliance on self-reported food-frequency questionnaires. Moreover, most studies assessed diet at a single time point at baseline, without accounting for dietary changes over time.

In conclusion, the current systematic review of RCTs and prospective cohort studies provides updated evidence on the association between adherence to MedDiet and the risk of T2D. Our findings suggest that higher adherence to MedDiet is inversely related to the risk of T2D, strengthened by a dose–response gradient in observational studies. The MedDiet is a healthy dietary pattern and could be useful in preventing T2D.

## Author contributions

The authors’ responsibilities were as follows – LS, SW: designed the research, extracted the data, evaluated the certainty of evidence and are the guarantors; LS, EK, GB, JS, SW: conducted the literature search and literature screening; EK, GB, JS, SW: assessed the risk of bias of the included publications; LS, GH, SW: analyzed the data and wrote the first draft of the article; and all authors: interpreted the data, read the manuscript, and approved the final version.

## Data availability

Data were extracted from published prospective observational studies. Results of unpublished data can be found in the Supplemental Material.

## Funding

The project itself had no funding source.

## Conflict of interest

LS is an associate editor of *Advances in Nutrition* Journal. Given his role as editor, he had no involvement in the peer review of this article and had no access to information regarding its peer review. Full responsibility for the editorial process for this article was delegated to another journal editor. The other authors declare that they have no known competing financial interests or personal relationships that could have appeared to influence the work reported in this article. All authors have completed the ICMJE uniform disclosure form at www.icmje.org/disclosure-of-interest/ and declare to have no conflict of interest.
