# Supplementary material for: Adherence to Mediterranean Diet and Risk of Type 2 Diabetes: An Updated Systematic Review and Dose–Response Meta-analysis
Source: Adv Nutr. 2025 Nov 13;16(12):100562. doi: 10.1016/j.advnut.2025.100562 (PMC12720176; doi:10.1016/j.advnut.2025.100562)
Supplement: Multimedia component 1 [file mmc1.docx]

**Adherence to Mediterranean diet and risk of type 2 diabetes: an updated systematic review and dose-response meta-analysis**

Sabina Wallerer^1^, Julia Stadelmaier^1^, Eike Floegel^1^, Eva Kiesswetter^1^, Gina Bantle^1^, Georg Hoffmann^2^, Lukas Schwingshackl^1^

**Data supplement**

**Table of Contents**

[Supplemental Table 1: Eligibility criteria by the PI/ECOS statement 3](#_Toc206480350)

[Supplemental Table 2: List of excluded studies 3](#_Toc206480351)

[Supplemental Table 3: Publications excluded due to overlap 4](#_Toc206480352)

[Supplemental Table 4: General characteristics of the included studies for the outcome Type 2 diabetes mellitus 5](#_Toc206480353)

[Supplemental Table 5: General characteristics of the included studies for the outcome Type 2 diabetes mellitus, including Score (components, range), Results, Covariable adjustments 9](#_Toc206480354)

[Supplemental Figure 1: Risk of bias of the included randomized controlled trial 19](#_Toc206480355)

[Supplemental Figure 2: Risk of bias of included cohort studies 20](#_Toc206480356)

[Supplemental Figure 3: Forest plot for the association of Mediterranean Diet Adherence and Type 2 diabetes mellitus by MedDiet score 21](#_Toc206480357)

[Supplemental Figure 4: Forest plot for the association of Mediterranean Diet Adherence and Type 2 diabetes mellitus by risk of bias judgement 22](#_Toc206480358)

[Supplemental Figure 5: Forest plot for the association of Mediterranean Diet Adherence and Type 2 diabetes mellitus excluding EPIC-Norfolk and EPIC-Potsdam 23](#_Toc206480359)

[Supplemental Figure 6: Funnel Plot for the association of Mediterranean Diet Adherence and Type 2 diabetes mellitus 24](#_Toc206480360)

[Supplemental Appendix 1: Search strategy 25](#_Toc206480361)

[Supplemental Appendix 2: Additional description and decision criteria for each domain in ROB 2 assessment 27](#_Toc206480362)

[Supplemental Appendix 3: Detailed description and decision criteria for each domain in ROBINS-E assessment 31](#_Toc206480363)

[Supplemental References 39](#_Toc206480364)

# Supplemental Table 1: Eligibility criteria by the PI/ECOS statement

|  | **Inclusion criteria** | **Exclusion criteria** |
| --- | --- | --- |
| **P** (population) | Adults (aged ≥18 years), generally healthy population | Study populations with a particular condition such as pre-diabetes, metabolic dysfunction–associated steatotic liver disease, chronic kidney disease, or cancer; studies involving exclusively infants, children, adolescents, or pregnant women |
| **I/E** (intervention/exposure)  **C** (comparison) | Studies evaluating the association of an a priori score used for assessing adherence to a MedDiet (e.g., traditional MedDiet or the alternate MedDiet score).  For RCTs: MedDiet vs. any other diet (e.g., low fat diet) | A posteriori score using principal component analysis or cluster analysis |
| **O** (outcome) | Type 2 diabetes mellitus | All other outcomes |
| **S** (study design) | Randomized controlled trials, Prospective observational studies (e.g., nested case-control study, case-cohort study) | Cross-sectional and retrospective studies |

PI/ECOS, Population, Intervention/Exposure, Comparison, Outcome, Study design; MedDiet, Mediterranean Diet; RCT, randomized controlled trial,

# Supplemental Table 2: List of excluded studies

| **Reason for exclusion** | **References** |
| --- | --- |
| Wrong intervention/exposure | (1-5) |
| Wrong patient population | (6-10) |
| Wrong outcome | (11, 12) |
| Wrong study design | (13) |
| Suspected fraudulent study | (14) |

# Supplemental Table 3: Publications excluded due to overlap

| **Excluded due to Overlap** | **Included** | **Cohort** |
| --- | --- | --- |
| André 2020 (15), Maroto-Rodriguez 2023 (16) | Fan 2024 (17) | UK Biobank |
| Aryannezhad 2025 (18) | Wang 2024 (19) | EPIC-Norfolk |
| Cespedes 2016 (20) | Glenn 2023 (21) | WHI |
| Kachegia 2024 (22), Koloverou 2016 (23) | Damigou 2025 (24) | ATTICA |
| Eguaras 2017 (25) | Ruiz-Estigarribia 2020 (26) | SUN |
| Khalili-Moghadam 2019 (27), Ramezan 2019 (28) | Esfandiar 2022 (29) | TLGS |
| Jannasch 2024 (30) | Galbete 2018 (31) | EPIC-Potsdam |
| Jacobs 2017 (32) | Jacobs 2015 (33) | MEC |
| Martinez-Gonzales 2008 (34), Martinez-Gonzales 2023 (35) | Salas-Salvado (36) | PREDIMED |
| Freisling 2020 (37) | Romaguera 2011 (38) | EPIC-InterAct |

ATTICA, ATTICA study; EPIC The European Prospective Investigation into Cancer and Nutrition; MEC, The Multiethnic Cohort; PREDIMED, Prevención con Dieta Mediterránea; SUN, Seguimiento Universidad de Navarra; TGLS, Teheran Lipid and Glucose Study; WHI, Women’s Health Initiative

# Supplemental Table 4: General characteristics of the included studies for the outcome Type 2 diabetes mellitus

| Author and year | Cohort and location | Population and health status | Sex | Age at entry (years) | BMI | *n*  cases | Outcome and criteria | Exposure assessment | Follow-up (years) |
| --- | --- | --- | --- | --- | --- | --- | --- | --- | --- |
| **Cohort studies** | | | | | | | | | |
| Abiemo 2013(39) | MESA (USA) | *n* 5390  generally healthy | M/W | 62 | 27.9 | 412 | T2DM  FBG ≥ 126 mg/dl, self-report of diagnosis or use of antidiabetic medication | Validated FFQ  single assessment | 6 |
| Ahmad 2020(40) | WHS (USA) | *n* 25317  generally healthy | W | 52.9 | NR | 2307 | T2DM  self-report of diagnosis, verified by telephone interview or supplemental questionnaire | Validated FFQ  single assessment | 19.8 |
| Bantle 2016(41) | CARDIA (USA) | *n* 3358  generally healthy | M/W | 25 | 24.4 | 393 | T2DM  FBG ≥ 126 mg/dl, 2h-OGTT ≥ 200 mg/dl, HbA1c ≥ 6.5% or self-reported use of antidiabetic medication | Validated DHQ  single assessment | 25 |
| Chen 2018(42) | SCHS (Asia) | *n* 45411  generally healthy | M/W | 55.2 | 23 | 5207 | T2DM  self-report of diagnosis | Validated FFQ  single assessment | 11.1 |
| Crawford 2023(43) | Sister Study (USA) | *n* 40243  generally healthy | W | 55.2 | 27.1 | 2486 | T2DM  self-report of diagnosis, use of oral antidiabetic medication or insulin | Validated FFQ  single assessment | 11.6 |
| de Koning 2011(44) | HPFS (USA) | *n* 41615  generally healthy | M | 40-75 | 25.4 | 2795 | T2DM  self-report of diagnosis or use of antidiabetic medication | Validated FFQ  repeated assessment | 17.6 |
| de León 2012(45) | CDC de Canarias (Europe) | *n* 5521  generally healthy | M/W | 42 | NR | 146 | T2DM  self-report of diagnosis, review of medical records | Validated FFQ  single assessment | 3.5 |
| Esfandiar 2022(29) | TLGS (Asia) | *n* 6112  generally healthy | M/W | 41.2 | 27.1 | 549 | T2DM  FBG ≥ 126 mg/dl, 2h-OGTT ≥ 200 mg/dl or use of antidiabetic medication | Validated FFQ  single assessment | 6.6 |
| Fan 2024(17) | UK Biobank (Europe) | *n* 78230  generally healthy | M/W | 57.7 | 29.2 | 1693 | T2DM  Linkage to medical records | 24 h recall  repeated assessment | 12.2 |
| Galbete 2018(31) | EPIC-Potsdam (Europe) | *n* 23411  generally healthy | M/W | 49.8 | 26.1 | 1376 | T2DM  Self-report of diagnosis, medical records, record linkage | Validated FFQ  single assessment | 10.5 |
| Glenn 2023(21) | WHI (USA) | *n* 145299  generally healthy | W | 63.1 | 27.7 | 13943 | T2DM  self-report of diagnosis, use of oral antidiabetic medication or insulin | Validated FFQ  mixed assessment (repeated assessment for women participating in observational study) | 16 |
| Hlaing-Hlaing 2021(46) | ALSWH (Australia) | *n* 3905  generally healthy | W | 52.5 | NR | 375 | T2DM  self-report of diagnosis | Validated FFQ  repeated assessment (only the first was included in the analysis) | 15 |
| Hodge 2021(47) | MCCS (Australia) | *n* 39185  generally healthy | M/W | 55.2 | 26.8 | 1989 | T2DM  self-report of diagnosis | Validated FFQ  single assessment | 13 |
| Jacobs 2015(33) | MEC (USA) | *n* 89185  generally healthy | M/W | 58 | 25.1 | 11217 | T2DM  self-report of diagnosis, antidiabetic medication inventory or linkage with health insurance plans and diabetic care registries | Validated FFQ  single assessment | 6 |
| Damigou 2025(24) | ATTICA (Europe) | *n* 2000  generally healthy | M/W | 42 | 26 | 526 | T2DM  assessment by study physicians and review of medical records | Validated FFQ  repeated assessment | 20 |
| Lin 2023(48) | TwSHHH (Asia) | *n* 4705  generally healthy | M/W | 43.7 | 23.2 | 978 | T2DM  FPG ≥ 126 mg/dl, HbA1c ≥ 5.6%, linkage with medical records, at least 2 outpatient visits or 1 hospital admission with T2DM diagnosis, or use of antidiabetic medication | FFQ  single assessment | 5.3 |
| O`Connor 2020(49) | ARIC (USA) | *n* 11956  generally healthy | M/W | 53.8 | 27.3 | 4013 | T2DM  FBG ≥ 126 mg/dl, nFBG ≥ 200 mg/dl, self-report of diagnosis or use of antidiabetic medication | Validated FFQ  repeated assessment | 22 |
| Rai 2023(50) | MASALA (Asia) | *n* 735  generally healthy | M/W | 55.2 | 26.0 | 45 | T2DM  FPG ≥ 126 mg/dl, 2h-OGTT ≥ 200 mg/dl or self-reported use of antidiabetic medication | Validated FFQ  single assessment | 5 |
| Romaguera 2011^a^ (38) | EPIC-Interact (Europe) | *n* 340 234  generally healthy | M/W | 52.4 | 26.0 | 11994 | T2DM  self-report of diagnosis and linkage to primary/secondary care registers, drug registers, hospital admissions and mortality data | Validated FFQ  single assessment | *4-million-person years* |
| Rossi 2013(51) | EPIC-Greece (Europe) | *n* 22 295  generally healthy | M/W | 50.2 | 28.0 | 2330 | T2DM  self-report of diagnosis or use of antidiabetic medication and review of medical records, discharge diagnosis or death certificates | Validated FFQ  single assessment | 11.3 |
| Tison 2022(52) | REGARDS (USA) | *n* 8750  generally healthy | M/W | 63.2 | NR | 1026 | T2DM  FBG ≥ 126 mg/dl, RBG ≥ 200 mg/dl or self-reported use of antidiabetic medication | Validated FFQ  single assessment | 10 |
| Ying 2024(53) | CHNS (Asia) | *n* 12575  generally healthy | M/W | 42.5 | 22.3 | 445 | T2DM  self-report of diagnosis | 24 h recall  repeated assessment | 9 |
| Wang 2024(19) | EPIC-Norfolk (Europe) | *n* 21900  generally healthy | M/W | 58.6 | 26.2 | 1662 | T2DM  review of hospital admission records | Validated FFQ  single assessment | 21.4 |
| Ruiz-Estigarribia 2020(26) | SUN (Europe) | *n* 11005  generally healthy | M/W | 40.2 | 25.5 | 145 | T2DM  self-report of diagnosis or use of antidiabetic medication | Validated FFQ  single assessment | 12 |
| **RCTs** | | | | | | | | | |
| Salas-Salvado 2014(36) | PREDIMED (Europe) | *n* 3541  high risk of CVD | M/W | 66.6 | 30.0 | 273 | T2DM  FPG ≥ 126 mg/dl, 2h-OGTT ≥ 200 mg/dl and annual review of medical records | Validated FFQ  repeated assessment | 4.1 |

^a^EPIC InterAct is a case-cohort study. Romaguera 2011 included 15049 controls and 11994 cases into the analysis. The complete number of participants was extracted from Forouhi 2014 (54).

2h-OGTT 2-hour, oral glucose tolerance test; ALSWH, The Australian Longitudinal Study on Women’s Health; ARIC, The Atherosclerosis Risk in Communities Study; BMI, body mass index; CARDIA, The Coronary Artery Risk Development in Young Adults Study; CDC, Cardiovascular, Diabetes, Cáncer; CHNS, The China Health and Nutrition Survey; CVD, cardiovascular disease; DHQ, diet history questionnaire; EPIC, The European Prospective Investigation into Cancer and Nutrition; FBG, fasting blood glucose; FFQ, food frequency questionnaire; FPG, fasting plasma glucose; HbA1c, glycated hemoglobin type A; HPFS, The Health Professionals Follow-Up Study; MASALA, The Mediators of Atherosclerosis in South Asians Living in America Study; MCCS, The Melbourne Collaborative Cohort Study; MD, Mediterranean diet; MEC, The Multiethnic Cohort; MESA, Multi-Ethnic Study of Atherosclerosis; nFBG, non-fasting blood glucose; NR, not reported; PREDIMED, Prevención con Dieta Mediterránea; RBG, random blood glucose; RCT, randomized controlled trial; REGARDS, The REasons for Geographic And Racial Differences in Stroke Study; SAM, South Asian Mediterranean-style diet score; SBP, systolic blood pressure; SCHS, The Singapore Chinese Health Study; SUN, Seguimiento Universidad de Navarra; T2DM, type 2 diabetes mellitus; TLGS, Teheran Lipids and Glucose Study; TwSHHH, The Taiwanese Survey on Prevalence of Hypertension, Hyperglycaemia and Hyperlipidaemia; UK, United Kingdom; USA, United States of America; WHI, Women`s Health Initiative; WHS, Women`s Health Study

# Supplemental Table 5: General characteristics of the included studies for the outcome Type 2 diabetes mellitus, including score (components, range), results, covariable adjustments

| Author and year | Mediterranean Diet Score and components | Range | Results | | | Covariable adjustments |
| --- | --- | --- | --- | --- | --- | --- |
|  |  |  | Measure | Score | Effect estimate  (95 % CI) |  |
| Cohorts | | | | | | |
| Abiemo 2013(39) | aMED  ↑ Vegetables (except potatoes)  ↑ Legumes  ↑ Fruit  ↑ Nuts  ↑ Whole grains  ↑ Fish  ↑ MUFA: SFA  ↓ Red meat  ↓ Dairy products  ↔ Alcohol (>5 g/d and <15 g/d) | 0-10 | HR | 1.5 | Ref. | Age, sex, energy, WC, smoking, education, income, physical activity, ethnicity, study site |
|  |  |  |  | 4 | 1.01 (0.74, 1.38) |  |
|  |  |  |  | 5 | 1.04 (0.77, 1.41) |  |
|  |  |  |  | 6 | 1.08 (0.78, 1.50) |  |
|  |  |  |  | 8.5 | 1.09 (0.80, 1.49) |  |
|  |  |  |  | per 1-quintile increase | 1.02 (0.95, 1.10) |  |
| Ahmad 2020(40) | aMED  ↑ Vegetables (except potatoes)  ↑ Legumes  ↑ Fruit  ↑ Nuts  ↑ Whole grains  ↑ Fish  ↑ MUFA: SFA  ↓ Red and processed meat  ↔ Alcohol (>5 g/d and <15 g/d) | 0-9 | HR | 1.5 | Ref. | Age, energy, smoking, physical activity, randomized treatment assignment, menopausal status, postmenopausal hormone use |
|  |  |  |  | 4.5 | 1.00 (0.91, 1.10) |  |
|  |  |  |  | 7.5 | 0.80 (0.71, 0.90) |  |
| Bantle 2016(41) | amMED  ↑ Vegetables  ↑ Legumes  ↑ Fruit & nuts  ↑ Fish & seafood  ↑ Whole grains  ↑ Eggs  ↑ Milk  ↑ (MUFA+PUFA)/ SFA  ↓ Meat and poultry  ↓ Refined grains  ↓ Potatoes  ↓ Snacks  ↓ Sweets  ↓ Beverages  ↔ Alcohol (10-50g for men and 5-25g for women) | 0-15 | OR | per 2.1 score points increase | 0.90 (0.79, 1.03) | Age, sex, energy, BMI, smoking, education, physical activity, race, field center |
| Chen 2018(42) | aMED  ↑ Vegetables (except potatoes)  ↑ Legumes  ↑ Fruit  ↑ Nuts  ↑ Whole grains  ↑ Fish  ↑ MUFA: SFA  ↓ Red and processed meat  ↔ Alcohol (>5 g/d and <15 g/d) | 0-9 | HR | 2 | Ref. | Age, sex, energy, BMI, smoking, education, coffee, physical activity, hypertension, dialect group, year of baseline interview |
|  |  |  |  | 3 | 0.96 (0.88, 1.05) |  |
|  |  |  |  | 4 | 0.92 (0.84, 1.00) |  |
|  |  |  |  | 5 | 0.83 (0.76, 0.91) |  |
|  |  |  |  | 6 | 0.84 (0.77, 0.92) |  |
|  |  |  |  | per SD increase (1.68 score points) | 0.93 (0.91, 0.96) |  |
| Crawford 2023(43) | aMED  ↑ Vegetables (except potatoes)  ↑ Legumes  ↑ Fruit  ↑ Nuts  ↑ Whole grains  ↑ Fish  ↑ MUFA: SFA  ↓ Red and processed meat  ↔ Alcohol (>5 g/d and <15 g/d) | 0-9 | HR | 1 | Ref. | Age (primary time scale, and thus, not included as a covariate in the multivariable models), energy, smoking, education, income, physical activity, vitamin/supplement use, race, menopausal status, hormone therapy, hormonal contraceptive use, family history of type 2 diabetes, area deprivation index |
|  |  |  |  | 3.5 | 0.93 (0.84, 1.03) |  |
|  |  |  |  | 5 | 0.80 (0.70, 0.92) |  |
|  |  |  |  | 7 | 0.66 (0.58, 0.75) |  |
|  |  |  |  | per 1-SD increase | 0.84 (0.80, 0.88) |  |
| de Koning 2011(44) | aMED  ↑ Vegetables (except potatoes)  ↑ Legumes  ↑ Fruit  ↑ Nuts  ↑ Whole grains  ↑ Fish  ↑ MUFA: SFA  ↓ Red and processed meat  ↔ Alcohol (>5 g/d and <15 g/d) | 0-9 | HR | 1 | Ref. | Energy, BMI, smoking, coffee, physical activity, family history of type 2 diabetes |
|  |  |  |  | 3 | 0.92 (0.82, 1.03) |  |
|  |  |  |  | 4 | 0.91 (0.81, 1.02) |  |
|  |  |  |  | 5.5 | 0.89 (0.79, 1.00) |  |
|  |  |  |  | 8 | 0.75 (0.66, 0.86) |  |
|  |  |  |  | per SD increase (2 score points) | 0.91 (0.87, 0.96) |  |
| de León 2012(45) | tMED  ↑ Vegetables  ↑ Legumes  ↑ Fruit & Nuts  ↑ Cereals  ↑ Fish  ↑ MUFA: SFA  ↓ Meat & Poultry  ↓ Dairy products  ↔ Alcohol (10-50 g/d for men and 5-25 g/d for women) | 0-9 | HR | Low (1.5) | Ref. | Age, sex |
|  |  |  |  | High (7.5) | 1.10 (0.70, 1.70) |  |
| Esfandiar 2022(29) | tMED  ↑ Vegetables  ↑ Legumes  ↑ Fruit & Nuts  ↑ Cereals  ↑ Fish  ↑ MUFA: SFA  ↓ Meat & Poultry  ↓ Dairy products | 0-8 | HR | 3 | Ref. | Age, sex, energy, smoking, fiber, physical activity, diabetes risk score |
|  |  |  |  | 4 | 1.02 (0.82, 1.26) |  |
|  |  |  |  | 5 | 0.89 (0.63, 1.24) |  |
|  |  |  |  | 6 | 1.06 (0.87, 1.30) |  |
| Fan 2024(17) | aMED  ↑ Vegetables (excluding potatoes)  ↑ Legumes  ↑ Fruit  ↑ Nuts  ↑ Whole grains  ↑ Fish  ↑ MUFA: SFA  ↓ Red and processed meat  ↔ Alcohol (>5 g/d and <15 g/d) | 0-9 | HR | 1.5 | Ref. | Age, sex, energy, smoking, employment status, education, household income, physical activity, ethnicity, polygenetic risk score |
|  |  |  |  | 4.5 | 0.95 (0.84, 1.08) |  |
|  |  |  |  | 7.5 | 0.68 (0.59, 0.77) |  |
| Galbete 2018(31) | tMED  ↑ Vegetables  ↑ Legumes  ↑ Fruit & Nuts  ↑ Cereals  ↑ Fish  ↑ Olive oil  ↓ Meat & Poultry  ↓ Dairy products  ↔ Alcohol (10-50 g/d for men and 5-25 g/d for women) | 0-18 | HR | 3.5 | Ref. | Age, sex, energy, BMI, smoking, education, vitamin supplementation, cycling, sports, prevalent hypertension, WC |
|  |  |  |  | 9 | 0.92 (0.81, 1.04) |  |
|  |  |  |  | 14.5 | 0.84 (0.73, 0.97) |  |
|  |  |  |  | per 1 score point increase | 0.97 (0.95, 0.99) |  |
|  |  |  |  | per SD increase | 0.93 (0.88, 0.98) |  |
| Glenn 2023(21) | aMED  ↑ Vegetables  ↑ Legumes  ↑ Fruit  ↑ Nuts  ↑ Whole grains  ↑ Fish  ↑ MUFA: SFA  ↓ Red and processed meat  ↔ Alcohol (>5 g/d and <15 g/d) | 0-9 | HR | 2 | Ref. | Age, energy, smoking, alcohol, education, physical activity, hysterectomy history, hypertension, family history of diabetes, hormone therapy, cholesterol-lowering medication use, region, study arm, self-identified race and ethnicity, marital status |
|  |  |  |  | 3 | 0.96 (0.90, 1.01) |  |
|  |  |  |  | 4 | 0.92 (0.87, 0.97) |  |
|  |  |  |  | 5 | 0.87 (0.82, 0.92) |  |
|  |  |  |  | 6.5 | 0.78 (0.74, 0.83) |  |
|  |  |  |  | per SD increase | 0.90 (0.89, 0.93) |  |
| Hlaing-Hlaing 2021(46) | tMED  ↑ Vegetables  ↑ Legumes  ↑ Fruit & Nuts  ↑ Cereals  ↑ Fish  ↑ MUFA+PUFA:SFA  ↓ Meat & meat products  ↓ Dairy products  ↔ Alcohol (10-50 g/d for men and 5-25 g/d for women) | 0-9 | OR | Low (1.5) | Ref. | Age, smoking, SES, physical activity, taking prescribed and over-the-counter medicine, history of depression and/or anxiety at any previous survey(s) |
|  |  |  |  | High (7.5) | 0.76 (0.48, 1.21) |  |
| Hodge 2021(47) | tMED  ↑ Vegetables  ↑ Legumes  ↑ Fruit & Nuts  ↑ Cereals  ↑ Fish  ↑ MUFA+PUFA:SFA  ↓ Meat & meat products  ↓ Dairy products  ↔ Alcohol (10-50 g/d for men and 5-25 g/d for women) | 0-9 | IRR | 1.5 | Ref. | Age, sex, smoking, drinking status, SEIFA, physical activity, family history of diabetes |
|  |  |  |  | 5 | 0.93 (0.85, 1.02) |  |
|  |  |  |  | 8 | 0.97 (0.84, 1.13) |  |
| Jacobs 2015(33) | aMED  ↑ Vegetables (excluding potatoes)  ↑ Legumes  ↑ Fruit  ↑ Nuts  ↑ Whole grains  ↑ Fish  ↑ MUFA: SFA  ↓ Red and processed meat  ↔ Alcohol (>5 g/d and <15 g/d women 10-25 g/d men) | 0-9 | HR | Men: | Men: | Age, energy, BMI, smoking, education, physical activity, ethnicity |
|  |  |  |  | 1 | Ref. |  |
|  |  |  |  | 3 | 0.99 (0.91, 1.08) |  |
|  |  |  |  | 4 | 0.92 (0.84, 1.00) |  |
|  |  |  |  | 5.5 | 0.87 (0.80, 0.95) |  |
|  |  |  |  | 8 | 0.89 (0.80, 0.99) |  |
|  |  |  |  | per ratio of aMED/SD increase | 0.95 (0.92, 0.98) |  |
|  |  |  |  | Women: | Women: |  |
|  |  |  |  | 1 | Ref. |  |
|  |  |  |  | 3 | 0.95 (0.87, 1.04) |  |
|  |  |  |  | 4 | 1.02 (0.93, 1.11) |  |
|  |  |  |  | 5.5 | 0.96 (0.88, 1.06) |  |
|  |  |  |  | 7.5 | 0.92 (0.84, 1.02) |  |
|  |  |  |  | per ratio of aMED/SD increase | 0.97 (0.94, 1.00) |  |
| Damigou 2025(24) | MDS  ↑ Vegetables  ↑ Legumes  ↑ Fruit  ↑ Non-refined cereals  ↑ Fish  ↑ Olive oil  ↑ Potatoes  ↓ Meat and meat products  ↓ Poultry  ↓ Full-fat dairy products  ↔ Alcohol (300-700 ml/d) | 0-55 | OR | per 1 score point increase | 0.76 (0.59, 0.99) | Age, sex, BMI, smoking, SES, physical activity, medical history |
| Lin 2023(48) | aMED  ↑ Vegetables  ↑ Legumes  ↑ Fruit  ↑ Rice, noodles  ↑ Fish  ↓ Meat or poultry, hamburger | 0-6 | HR | 1.5 | Ref. | Age, sex, BMI, WC, smoking, alcohol, education, income, physical activity, sedentary time, SBP, family history of diabetes, marital status |
|  |  |  |  | 3 | 0.93 (0.78, 1.10) |  |
|  |  |  |  | 4 | 0.92 (0.78, 1.09) |  |
|  |  |  |  | 5.5 | 0.73 (0.59, 0.91) |  |
| O`Connor 2020(49) | aMED  ↑ Vegetables (excluding potatoes)  ↑ Legumes  ↑ Fruit  ↑ Nuts  ↑ Whole grains  ↑ Fish  ↑ MUFA: SFA  ↓ Red and processed meat  ↔ Alcohol (>5 g/d and <15 g/d women 10-25 g/d men) | 0-9 | HR | 1 | Ref. | Age, sex, energy, smoking, education, physical activity, race, study center |
|  |  |  |  | 3.5 | 0.99 (0.91, 1.08) |  |
|  |  |  |  | 5 | 0.93 (0.84, 1.03) |  |
|  |  |  |  | 6 | 0.88 (0.79, 0.99) |  |
|  |  |  |  | 8 | 0.88 (0.77, 0.99) |  |
|  |  |  |  | per 1 score point increase | 0.97 (0.96, 0.99) |  |
| Rai 2023(50) | saMED  ↑ Vegetables (excluding potatoes)  ↑ Legumes  ↑ Fruit  ↑ Nuts  ↑ Whole grains  ↑ Fish  ↑ MUFA: SFA  ↓ Red and processed meat  ↔ Alcohol (5-25 g/d) | 0-9 | OR | per 1 score point increase | 0.75 (0.59, 0.95) | Age, sex, energy, BMI, smoking, education, income, physical activity, years lived in the United States, perception of discrimination, the traditional cultural beliefs scale, family history of diabetes, cholesterol-lowering medication, antihypertensive medication |
| Romaguera 2011^a^(38) | rMED  ↑ Vegetables  ↑ Legumes  ↑ Fruit & Nuts  ↑ Cereals  ↑ Fish & Seafood  ↑ Olive oil  ↓ Meat & meat products  ↓ Dairy products  ↔ Alcohol (10-50 g/d for men and 5-25 g/d for women) | 0-18 | HR | 3 | Ref. | Age (time variable), sex, energy, BMI, smoking, education, physical activity, center (stratified) |
|  |  |  |  | 8.5 | 0.93 (0.86, 1.01) |  |
|  |  |  |  | 14.5 | 0.88 (0.79, 0.97) |  |
|  |  |  |  | per 2 score points increase | 0.96 (0.94, 0.99) |  |
| Rossi 2013(51) | tMED  ↑ Vegetables  ↑ Legumes  ↑ Fruit & Nuts  ↑ Cereals  ↑ Fish & Seafood  ↑ MUFA:SFA  ↓ Meat & meat products  ↓ Dairy products  ↔ Alcohol (10-50 g/d for men and 5-25 g/d for women) | 0-9 | HR | 1.5 | Ref. | Age, sex, energy, BMI, WHR, education, physical activity |
|  |  |  |  | 4 | 1.00 (0.89, 1.12) |  |
|  |  |  |  | 5 | 0.94 (0.84, 1.06) |  |
|  |  |  |  | 7.5 | 0.88 (0.78, 0.99) |  |
|  |  |  |  | per 2 score points increase | 0.94 (0.89, 0.99) |  |
| Tison 2022(52) | tMED  ↑ Vegetables  ↑ Legumes  ↑ Fruit  ↑ Cereals  ↑ Fish  ↑ MUFA:SFA  ↓ Meat  ↓ Dairy products  ↔ Alcohol (>0 and ≤7 drinkes/wk for women and >0 and ≤14 drinkes/wk for men) | 0-9 | RR | 1.5 | Ref. | Age, sex, energy, smoking, alcohol, education, income, physical activity, race, region |
|  |  |  |  | 3 | 0.99 (0.84, 1.16) |  |
|  |  |  |  | 4 | 0.99 (0.84, 1.16) |  |
|  |  |  |  | 5 | 0.93 (0.77, 1.11) |  |
|  |  |  |  | 7.5 | 1.03 (0.84, 1.26) |  |
| Ying 2024(53) | tMED  ↑ Vegetables  ↑ Legumes  ↑ Fruit & Nuts  ↑ Cereals  ↑ Fish & Seafood  ↑ MUFA:SFA  ↓ Meat & meat products  ↓ Dairy products  ↔ Alcohol (10-50 g/d for men and 5-25 g/d for women) | 0-9 | HR | 1.5 | Ref. | Age, sex, energy, BMI, smoking, occupations, education, income, physical activity, region, urban or rural residents |
|  |  |  |  | 3 | 0.69 (0.54, 0.89) |  |
|  |  |  |  | 4 | 0.65 (0.50, 0.86) |  |
|  |  |  |  | 6.5 | 0.48 (0.34, 0.69) |  |
|  |  |  |  | per 1 score point increase | 0.83 (0.76, 0.90) |  |
| Wang 2024(19) | tMED  ↑ Vegetables  ↑ Legumes  ↑ Fruit & Nuts  ↑ Cereals  ↑ Fish  ↑ MUFA(&PUFA):SFA  ↓ Meat & meat products  ↓ Dairy products  ↔ Alcohol (10-50 g/d for men and 5-25 g/d for women) | 0-9 | HR | 1.5 | Ref. | Age, sex, BMI, smoking, social-class, education, physical activity, marital status, medication use, family history of MI, stroke, or diabetes |
|  |  |  |  | 4 | 0.93 (0.81, 1.06) |  |
|  |  |  |  | 5 | 0.80 (0.70, 0.92) |  |
|  |  |  |  | 7.5 | 0.82 (0.72, 0.93) |  |
|  |  |  |  | per SD increase (1.78 score points) | 0.90 (0.85, 0.94) |  |
| Ruiz-Estigarribia 2020(26) | tMED  ↑ Vegetables  ↑ Legumes  ↑ Fruit & Nuts  ↑ Cereals  ↑ Fish  ↑ MUFA: SFA  ↓ Meat & Poultry  ↓ Dairy products | 0-8 | HR | 1.3 | Ref. | Age (stratified deciles), sex, energy, BMI, smoking, alcohol, binge drinking, adoption of special diets, physical activity, personal history of hypertension, hypertriglyceridemia, hypercholesterolemia status, marital status, family history of diabetes, TV watching, sleep, time with friends, time working, recruitment period |
|  |  |  |  | 6.8 | 0.70 (0.50, 0.99) |  |
| RCTs | | | | | | |
| Salas-Salvado 2014(36) | Low-fat diet vs. MedDiet (increased consumption of vegetables, fruits, legumes, nuts, fish, olive oil, recommended amount of white meat (avoid red meat), decreased amount of butter, fast foods, sweets, pastries, sugar-sweetened beverages, alcohol) | NA | HR | MedDiet vs. low-fat diet | 0.75 (0.56, 1.01) | Age, sex, energy, BMI, smoking, alcohol, education, adherence to MD, physical activity, dyslipidemia, hypertension, fasting glucose level, center, propensity scores + exclusion of center D and second household members |

^a^EPIC InterAct is a case-cohort study. Romaguera 2011 included 15049 controls and 11994 cases into the analysis. The complete number of participants was extracted from Forouhi 2014 (54).

aMED, alternate Mediterranean diet score; amMED, Americanized Mediterranean Diet Score; BMI, body mass index; CI, confidence interval; HR, hazard ratio; IRR, incidence rate ratio; MedDiet, Mediterranean diet; MDS, Mediterranean diet score; MI, myocardial infarction; MUFA, monounsaturated fatty acids; NA, not applicable; OR, odds ratio; PUFA, polyunsaturated fatty acids; RCT, randomized controlled trial; Ref., reference; rMED, relative Mediterranean diet score; saMED, South Asian Mediterranean-style diet score; SBP, systolic blood pressure; SD, standard deviation; SEIFA, Socio-Economic Indexes for Areas; SES, socioeconomic status; SFA, saturated fatty acids; tMED, traditional Mediterranean diet score; WC, waist circumference; WHR, waist-hip ratio

# Supplemental Figure 1: Risk of bias of the included randomized controlled trial


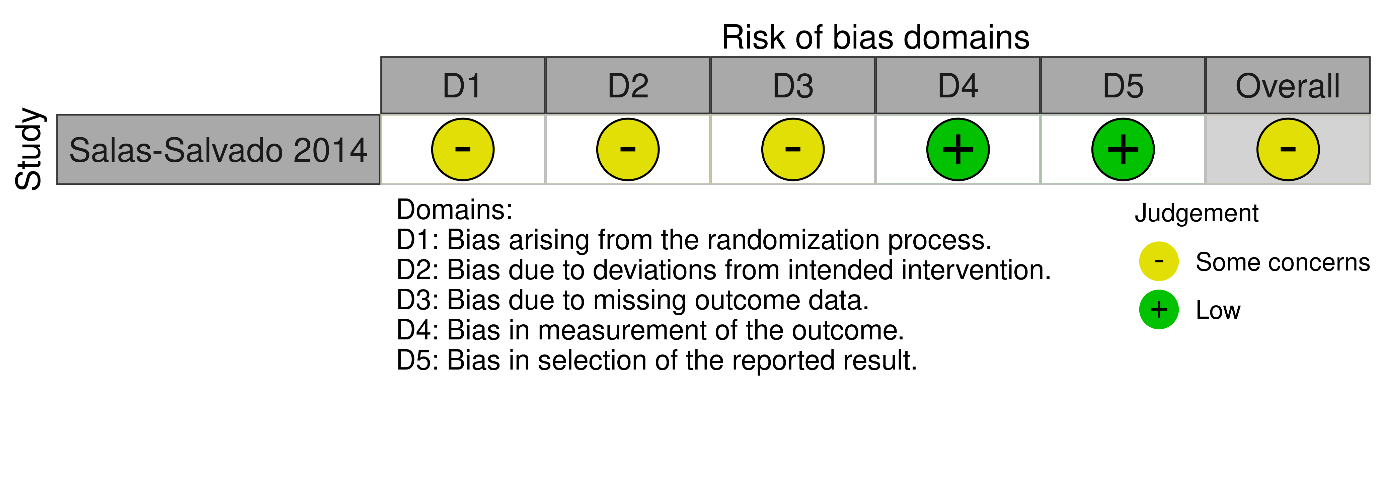


# Supplemental Figure 2: Risk of bias of included cohort studies


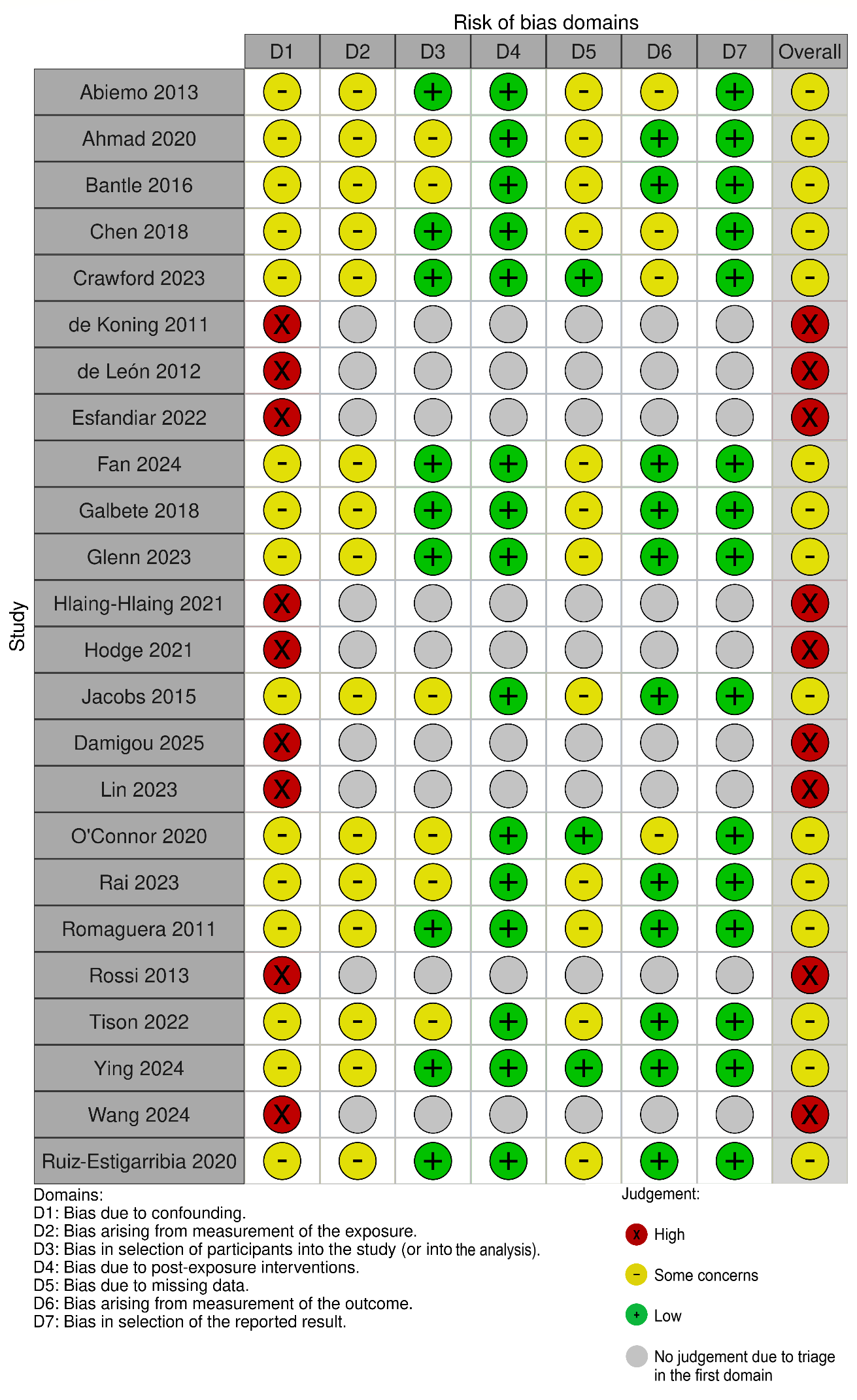


# Supplemental Figure 3: Forest plot for the association of Mediterranean Diet Adherence and Type 2 diabetes mellitus by MedDiet score


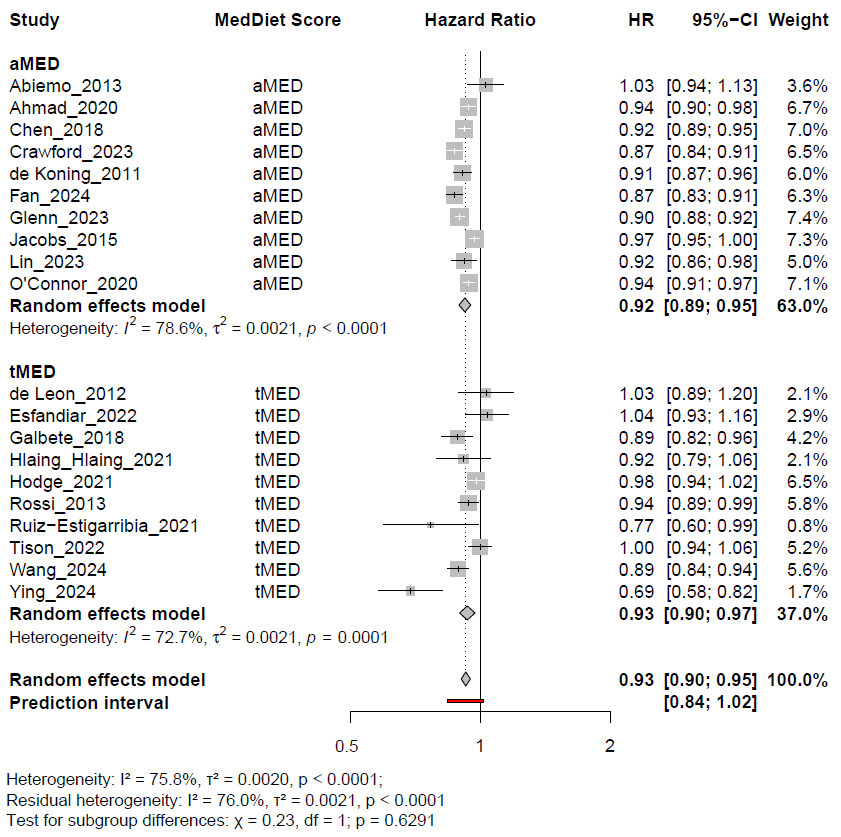


95% CI, 95% confidence Interval; aMED, alternate Mediterranean Diet Score; HR, hazard ratio; MedDiet, Mediterranean Diet; tMED, traditional Mediterranean Diet Score

# Supplemental Figure 4: Forest plot for the association of Mediterranean Diet Adherence and Type 2 diabetes mellitus by risk of bias judgement


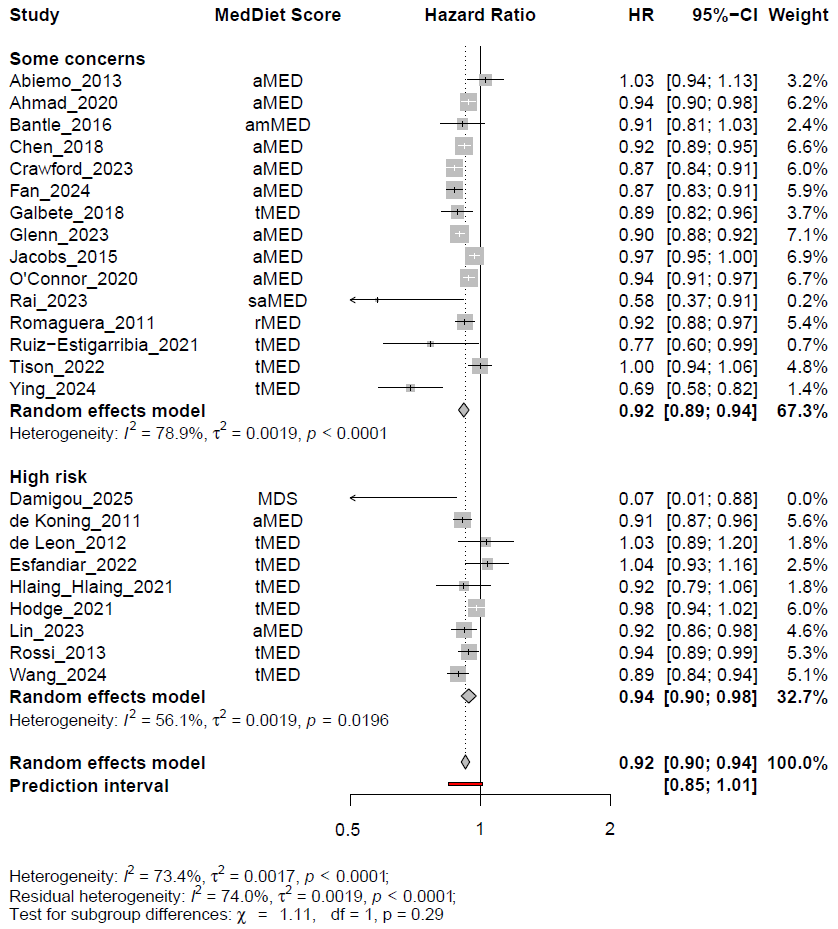


95% CI, 95% confidence Interval; aMED, alternate Mediterranean Diet Score; amMED, americanized Mediterranean Diet Score; HR, hazard ratio; MedDiet, Mediterranean Diet; MDS, Mediterranean Diet Score (modified); saMED, South Asian Mediterranean Diet Score; tMED, traditional Mediterranean Diet Score

# Supplemental Figure 5: Forest plot for the association of Mediterranean Diet Adherence and Type 2 diabetes mellitus excluding EPIC-Norfolk and EPIC-Potsdam


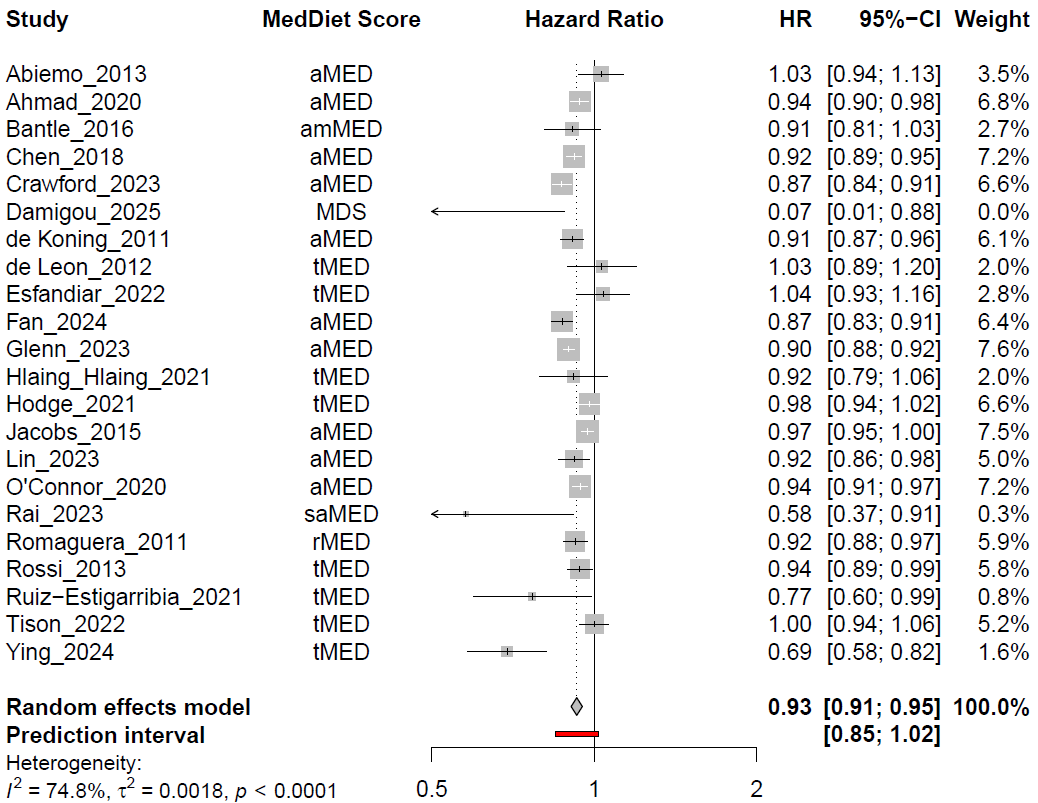


95% CI, 95% confidence Interval; aMED, alternate Mediterranean Diet Score; amMED, americanized Mediterranean Diet Score; HR, hazard ratio; MedDiet, Mediterranean Diet; MDS, Mediterranean Diet Score (modified); saMED, South Asian Mediterranean Diet Score; tMED, traditional Mediterranean Diet Score

# Supplemental Figure 6: Funnel Plot for the association of Mediterranean Diet Adherence and Type 2 diabetes mellitus


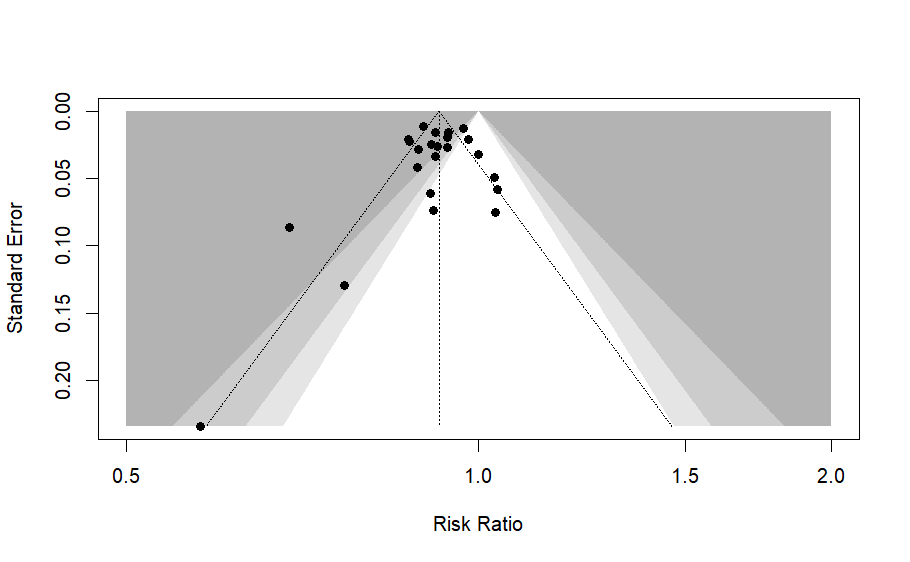


Funnel Plot of included cohort studies, excluding Damigou 2025 (24) due to having 0.0% weight in meta-analysis. P-value (Eggers linear regression test) = 0.55

# Supplemental Appendix 1: Search strategy

**Ovid MEDLINE® ALL** 1946 to May 23, 2025 (search: May 26, 2025)

|  | Search | Hits |
| --- | --- | --- |
| 1 | Diet, Mediterranean/ | 6,342 |
| 2 | (Mediterranean adj7 diet*).ti,ab,kf. | 10,890 |
| 3 | 1 or 2 | 11,557 |
| 4 | Diabetes Mellitus/ | 148,568 |
| 5 | Diabetes Mellitus, Type 2/ | 189,943 |
| 6 | (diabet* or T2D or T2DM).ti,ab,kf. | 874,675 |
| 7 | or/4-6 | 908,672 |
| 8 | 3 and 7 | 1,747 |
| 9 | exp randomized controlled trial/ | 640,958 |
| 10 | controlled clinical trial.pt. | 95,683 |
| 11 | (randomi?ed or placebo or randomly or trial or groups).ab. | 3,968,095 |
| 12 | or/9-11 | 4,109,861 |
| 13 | exp animals/ not humans.sh. | 5,340,475 |
| 14 | 12 not 13 | 3,572,648 |
| 15 | cohort studies/ or follow-up studies/ or longitudinal studies/ or prospective studies/ | 1,735,168 |
| 16 | (prospective or cohort* or observational or longitudinal or follow-up or cases or (case* and control*) or population-based).ti,ab,kf. | 5,356,756 |
| 17 | 15 or 16 | 5,847,282 |
| 18 | 8 and (14 or 17) | 1,004 |
| 19 | limit 8 to yr=”2014-Current” | **818** |

**Cochrane CENTRAL** May 26, 2025

|  | Search | Hits |
| --- | --- | --- |
| #1 | MeSH descriptor: [Diet, Mediterranean] this term only | 967 |
| #2 | (Mediterranean near/7 diet*):ti,ab,kw | 3,004 |
| #3 | #1 or #2 | 3,004 |
| #4 | MeSH descriptor: [Diabetes Mellitus] this term only | 12,750 |
| #5 | MeSH descriptor: [Diabetes Mellitus, Type 2] explode all trees | 26,570 |
| #6 | (diabet* or T2D or T2DM):ti,ab,kw | 127,028 |
| #7 | #4 or #5 or #6 | 127,028 |
| #8 | #3 and #7 | 654 |
|  | *with Publication Year from 2014 to present* | **532** |

**Scopus** May 26, 2025

|  | Search | Hits |
| --- | --- | --- |
| 1 | TITLE-ABS-KEY(Mediterranean W/7 diet*) | 18,651 |
| 2 | TITLE-ABS-KEY(diabet* or T2D or T2DM) | 1,419,408 |
| 3 | 1 AND 2 | 3,918 |
| 3 | TITLE-ABS-KEY ({Clinical-trial} OR {controlled-trial} OR randomi* OR randomly OR (random W/4 (allocat* OR distribut* OR assign*)) OR {placebo} OR {trial} OR {groups} OR {subgroups}) OR TITLE (rct) | 8,285,293 |
| 4 | TITLE-ABS-KEY(prospective or cohort* or observational or longitudinal or follow-up or cases or (case* and control*) or population-based) | 15,767,447 |
| 5 | 3 OR 4 | 21,721,986 |
| 6 | 3 AND 5 | 2,344 |
|  | 6 AND PUBYEEAR >2014 AND PUBYEAR <2026 | **1,907** |
|  | ((TITLE-ABS-KEY(mediterranean W/7 diet*)) AND (TITLE-ABS-KEY(diabet* OR t2d OR t2dm))) AND ((TITLE-ABS-KEY ({Clinical-trial} OR {controlled-trial} OR randomi* OR randomly OR (random W/4 (allocat* OR distribut* OR assign*)) OR {placebo} OR {trial} OR {groups} OR {subgroups}) OR TITLE (rct)) OR (TITLE-ABS-KEY(prospective OR cohort* OR observational OR longitudinal OR follow-up OR cases OR (case* AND control*) OR population-based)))  AND PUBYEAR > 2013 AND PUBYEAR < 2026 |  |

**Overview Search (May 26, 2025)**

| **Database** | **Hits** |
| --- | --- |
| Ovid MEDLINE(R) ALL | 818 |
| Cochrane CENTRAL | 532 |
| SCOPUS | 1,907 |
|  |  |
| **after deduplication** | **2,218** |

# Supplemental Appendix 2: Additional description and decision criteria for each domain in ROB 2 assessment

| **Domain 1: Risk of bias arising from randomisation process** | |
| --- | --- |
| **1.1** Was the allocation sequence random? | No information about randomisation method → NI |
| **1.2** Was the allocation sequence concealed until participants were enrolled and assigned to interventions? | Check if allocation was concealed (e.g. by using envelopes or a central or external enrolment service).  If appropriate allocation concealment can be assumed → PY/Y  If no information about allocation concealment is reported → NI |
| **1.3** Did baseline differences between intervention groups suggest a problem with the randomisation process? | Check group sizes.  Look for imbalances for key variables such as age, gender, health status, baseline values of outcomes.  Baseline tables:  If p-values are given, check for significant differences in baseline characteristics between intervention groups.  If p-values are not given, check (by eye) for large/obvious baseline imbalances between intervention groups.  Text: If small or no significant imbalances are reported → PN/N |

| **Domain 2: Risk of bias due to deviations from the intended interventions** | |
| --- | --- |
| **2.1** Were participants aware of their assigned intervention during the trial? | In studies with dietary interventions other than supplementation of vitamins/minerals, blinding is likely not possible due to the nature of the included interventions → Y/PY |
| **2.2** Were carers and people delivering the interventions aware of participants' assigned intervention during the trial? | In studies with dietary interventions other than supplementation of vitamins/minerals, blinding is likely not possible due to the nature of the included interventions → Y/PY |
| **2.3.** If Y/PY/NI to 2.1 or 2.2: Were there deviations from the intended intervention that arose because of the trial context? | Check if   1. additional interventions that were introduced were not consistent with trial protocol 2. failure to implement the protocol interventions as intended was evident   If no reasons or details of deviations from the planned interventions are reported, it is likely that no deviations occurred → PN/N  If reported deviations are expected to arise in usual care, e.g. disliked diet, missed visits, lost interest, difficulty following diet → PN/N |
| **2.4** If Y/PY to 2.3:  Were these deviations likely to have affected the outcome? | Judge whether the above mentioned aspects/deviations had an impact on the outcome. |
| **2.5** If Y/PY/NI to 2.4: Were these deviations from intended intervention balanced between groups? | See guidance |
| **2.6** Was an appropriate analysis used to estimate the effect of assignment to intervention? | If ITT or modified ITT was used → Y/PY  If ITT or modified ITT can be assumed (i.e. number randomised per group = number analysed per group) → Y/PY  If no details of the analysis are reported (i.e. number randomised per group ≠ number analysed per group, with no information about excluded participants) → NI  If per protocol analysis was used (with investigators actively excluding available data, e.g. due to reasons related to compliance) → PN/N |
| **2.7** If N/PN/NI to 2.6: Was there potential for a substantial impact (on the result) of the failure to analyse participants in the group to which they were randomised? | Cut-off: >5% missing per group (excluded or analysed in wrong group) |

| **Domain 3: Risk of bias due to missing outcome data** | |
| --- | --- |
| **3.1** Were data for this outcome available for all, or nearly all, participants randomised? | Note that imputed data should be regarded as missing data, and not considered as ‘outcome data’ in the context of this question.  Cut-off: ≥20% missing data → N/PN  Low RoB: <20% + valid reasons  Some concerns: <20% without valid reasons  However, if valid imputation techniques mentioned → low RoB |
| **3.2** If N/PN/NI to 3.1: Is there evidence that the result was not biased by missing outcome data? | Check if   1. (multiple) imputation was used 2. Sensitivity analysis were conducted 3. Reasons were given |
| **3.3** If N/PN to 3.2: Could missingness in the outcome depend on its true value?  **3.4** If Y/PY/NI to 3.3: Is it likely that missingness in the outcome depended on its true value? | High risk: > 20%  However, if:   - valid imputation techniques mentioned → low RoB - no imputation techniques are used, but valid reasons are mentioned for both groups and are (nearly) equally distributed across groups, we will not assume high RoB |
|  | |
| **Domain 4: Risk of bias in measurement of the outcome** | |
| **4.1** Was the method of measuring the outcome inappropriate? | - Check if self-reported outcomes were validated by a second source (register, medical records, second questionnaire). |
| **4.2** Could measurement or ascertainment of the outcome have differed between intervention groups? | Check if outcome measurement differed between groups.  If Y/PY → high RoB |
| **4.3** If N/PN/NI to 4.1 and 4.2: Were outcome assessors aware of the intervention received by study participants? | If N/PN → low RoB |
| **4.4** If Y/PY/NI to 4.3: Could assessment of the outcome have been influenced by knowledge of intervention received? | See guidance |
| **4.5** If Y/PY/NI to 4.4: Is it likely that assessment of the outcome was influenced by knowledge of intervention received? | See guidance |

| **Domain 5: Risk of bias in selection of the reported result** | |
| --- | --- |
| **5.1** Were the data that produced this result analysed in accordance with a pre-specified analysis plan that was finalised before unblinded outcome data were available for analysis?  Is the numerical result being assessed likely to have been selected, on the basis of the results, from...  **5.2.** ... multiple eligible outcome measurements (e.g. scales, definitions, time points) within the outcome domain?  **5.3** ... multiple eligible analyses of the data? | Check if   1. protocol or trial registry entry is available 2. information on the pre-specified analysis is given 3. changes to the pre-specific analysis plan were made (check also history of changes of the register entry) 4. deviations were reported in the manuscript.   If no study protocol/registration is available and no deviations are reported in the manuscript. → some concerns  If registry entry is available but no information about the analysis plan exists. → some concerns  If study protocol/registration is present and there is no evidence for differences between protocol and report. → low RoB  If study protocol/registration is present and differences between protocol and report were clearly described and justified in the text.  → low RoB  Cave: Only consider outcome pre-specification information that is dated before the end of trial. |

ITT: intention-to-treat analysis; NI: no information; PN/N: partial no/no; PY/Y: partial yes/yes; RoB: risk of bias

# Supplemental Appendix 3: Detailed description and decision criteria for each domain in ROBINS-E assessment

| **Domain** | **Explanation** | **Judgements** |
| --- | --- | --- |
| **Risk of bias due to confounding** | - Is there potential for confounding of the effect of exposure in this study? - Did the authors use a multivariable-adjusted analysis method that controlled at least for age, sex, energy, education/socioeconomic status, smoking, physical activity? - Were confounding factors that were controlled for measured validly and reliably by the variables available in this study? - Did the authors avoid adjusting for post-exposure variables?   *Notes:* Confounding is expected in all observational studies; thus, no study was assigned low risk of bias. Time-varying confounding was expected to be unlikely and is not expected to cause risk of bias in the present study. | Low risk of bias:  No bias is expected due to confounding, including time-varying confounding.  Some concerns:  Confounding is expected for age, sex, energy, education/socioeconomic status, smoking, physical activity, and the authors performed a multivariable-adjusted analysis to control for these confounding factors. The variables adjusted for are valid and reliable measures of the confounding factors.  *or*  Education/socioeconomic status (SES) is the only important covariate not included as confounding factor in the multivariable-adjusted analysis, but SES is not expected to vary substantially within the cohort (e.g. NHS, HPFS).  *or*  The authors statistically investigated whether the confounding factors have an effect on the risk estimate and excluded the confounder from the multivariable model if there was no effect on the overall effect estimate.  High risk of bias^a^:  At least one known important confounding factor was not measured or appropriately controlled for.  *or*  The authors adjusted for post-exposure variables that are affected by exposure (e.g. carbohydrate intake and risk of T2D [adjustment for BMI during follow-up = intermediate biological variable on the causal pathway] 🡪 over adjustment).  Very high risk of bias^a^:  No adjustment was made for any confounder.  *or*  The authors controlled for post-exposure variables, and the use of negative controls, or other considerations, suggest serious uncontrolled confounding. |
| **Risk of bias arising from measurement of exposure assessment** | - Does the measured exposure well-characterize the exposure metric specified to be of interest in this study? - Was the exposure likely to be measured with error, or misclassified?   *Notes:* Differential misclassification is not expected to occur in prospective cohort studies, since diet is reported before the occurrence of the outcome (Freedman 2011).  Some type of non-differential misclassification cannot be excluded (any dietary assessment method involves measurement error), thus no study was assigned low risk of bias. | Low risk of bias:  The exposure status is well characterised by the measurement and no measurement error is expected in its assessment.  *and*  The exposure was measured at multiple times, and is stable or changes only slightly over time.  Some concerns:  The exposure status is well characterised by the measurement, and was measured using an established or validated tool (e.g., a validated FFQ/DHQ, **multiple** 24h recalls).  *and*  The exposure was measured at multiple times, and it is stable or changes only slightly over time.  *or*  The exposure was measured by a single measurement assessing longer periods of time (i.e. validated FFQ/DHQ), and is therefore assumed to be stable over time.  High risk of bias:  The exposure status is not well characterized by the measurement (e.g., assumed from an indirect measurement or important sources of dietary intake are not considered).  *and/or*  The exposure was measured using a not validated tool.  *and/or*  The exposure was measured with a single measurement, which is unlikely to characterize the exposure over a longer period of time (e.g., single 24h recall) and therefore cannot be assumed to be representative.  *and/or*  The exposure cannot be assumed to be stable over time.  Very high risk of bias:  Differential measurement error is expected (measurement error depends on the outcome). |
| **Risk of bias in selection of participants into the study** | - Was selection of participants into the study (or into the analysis) based on participant characteristics observed after the start of the exposure window being studied? - Do start of follow-up and start of exposure coincide for most participants? - Were methods used that are likely to correct for the presence of selection biases?   Notes: In observational studies, it is unlikely that post-exposure variables influenced selection of participants into the study. Exclusion of participants may be mostly based on missing data, which will be considered in the domain referring to missings (see below). | Low risk of bias:  All participants who would have been eligible for the target study were included in the study.  *and*  The authors conducted a sensitivity analysis excluding type 2 diabetes cases which occurred <2 years after the start of the study and the results did not change*.*  *and*  The start of exposure and follow-up coincide.  Some concerns:  The selection into the study may have been related to exposure and outcome.  *and*  The authors used appropriate methods to correct for the selection bias.  *or*  The authors conducted no sensitivity analysis excluding cases of the respective outcome (e.g. CVD, mortality, type 2 diabetes) which occurred <2 years after start.  *and/or*  The start of exposure and follow-up do not coincide, but the association of exposure is constant over time.  High risk of bias:  The selection into the study was related to exposure and outcome.  *and/or*  The start of exposure and follow-up do not coincide and the effect of exposure is not constant over time.  *and*  This could not be corrected for in the analyses.  Very high risk of bias:  The selection into the study was related to exposure and outcome.  *and/or*  The start of exposure and follow-up do not coincide and the effect of exposure is not constant over time.  *and*  A sensitivity analysis is available that demonstrates substantial impact. |
| **Risk of bias due to post-exposure interventions** | - Were there post-exposure interventions that were influenced by prior exposure during the follow-up period?   *Notes:* In prospective observational studies, post-exposure interventions are unlikely. We don’t expect any issues in this domain for our analysis. | Low risk of bias:  There were (probably) no interventions administered to alleviate the effect of exposures.  Some concerns:  Post-exposure interventions were identified and the analysis corrected for the effect of these interventions.  High risk of bias:  Post-exposure interventions were identified and the analysis did not correct for the effect of these interventions |
| **Bias due to missing data** | - Were there missing outcome data? - Were participants excluded due to missing data on exposure status? - Were participants excluded due to missing data on other variables needed for analysis? - Did the authors perform a complete case analysis? - Was an appropriate method used to correct for bias due to missing data (e.g. appropriate imputation)?   *Notes:* Missing data on exposure variables and other variables are expected to be missing at random and not related to exposure or outcome that have been assessed during follow-up. | Low risk of bias:  There was little loss-to-follow-up (<20%) and data on exposure and other variables were reasonably complete (<10% missing data) and was unlikely to introduce bias.  *or*  The analysis addressed missing data and is likely to have removed any risk of bias.  Some concerns:  There is a proportion (>10%) of missing data in the original cohort or a high proportion (>20%) of loss-to-follow-up.  *and*  The analysis is unlikely to have removed the risk of bias arising from the missing data (e.g., using logistic regression).  *or*  There is a significant proportion (>20%) of missing data but the authors addressed this issue by appropriate methods (i.e. imputation of data).  High risk of bias:  There are high proportions (>50%) of missing data.  *and*  The analysis is unlikely to have removed the risk of bias arising from the missing data.  *or*  The nature of the missing data means that the risk of bias cannot be removed through appropriate analysis.  Very high risk of bias:  There are high proportions (>50%) of missing data; and missing data were addressed inappropriately in the analysis. |
| **Risk of bias due to measurement of the outcome** | - Were the methods of outcome assessment comparable across exposure groups? - Could the outcome measure have been influenced by knowledge of the exposure status? - Were any systematic error in measurement of the outcome related to exposure status?   *Notes:* In prospective observational studies, it is not expected that outcome assessors were aware of exposure status of the participants. | Low risk of bias:  The methods of outcome assessment were comparable across all exposure groups.  *and*  The outcome measure was unlikely to be influenced by knowledge of the exposure status of study participants.  *and*  Any error in measuring the outcome is unrelated to exposure status (i.e. objective measures or self-reported outcomes that are mostly (≥90%) confirmed by a second source, e.g. medical records, record linkage).  Some concerns:  The methods of the outcome assessment were comparable across exposure groups.  *and*  Any error in measuring the outcome may be minimally related to exposure status.  *or*  The measurement of the outcome is not reliable (i.e. confirmed records are available for <90% of all participants and the authors did not perform an additional analysis separating confirmed and probable cases).  High risk of bias:  The methods of outcome assessment were not comparable across exposure groups.  *or*  The outcome measure was subjective (i.e. self-report of type 2 diabetes by study participants or next of kin, without confirmation by a second source).  *and/or*  Any error in measuring the outcome was related to exposure status. |
| **Risk of bias due to selection of the reported result** | - Was the result reported in accordance with an available, pre-determined analysis plan? - Is the reported effect estimate likely to be selected from multiple exposure measurements? - Is the reported effect estimate likely to be selected from multiple analyses of exposure-outcome relationship? - Is the reported effect estimate likely to be selected from different subgroups?   *Notes:* In observational studies, it is unusual to publish an a priori analysis plan or protocol. Therefore, if the authors present a clear description of the conducted analyses (i.e. methods section), and it appears to be consistent with the reported results; and the reported results correspond to all intended outcomes, analyses and sub cohorts (e.g. postmenopausal women), low risk of bias can be adequate.  However, if there are any inconsistencies/or no information between intended analyses and reported results, e.g.:   - a substitution analysis was conducted in line with the methods section, but reported results were incomplete (e.g. authors state that a substitution analysis for fat vs. carbohydrates was conducted, but estimates are not reported); - results of substitution analyses reported, but methodological approach not described; - there are inconsistencies between the adjustments described in the methods section and the adjustments for the corresponding reported estimates in the results section;   there is reason for some concerns in this domain.  Multiple outcome measurements for the definition of CVD, mortality, type 2 diabetes, etc. are not expected. | Low risk of bias:  The results are reported according to an a-priori analysis plan or protocol or there is a clear description of all analysis, the analyses are consistent, and all reported results correspond to all intended outcomes, analyses and sub-cohorts.  Some concerns:  There is an a-priori analysis plan or protocol available, and there is indication of selection of the reported analysis among multiple analyses; or there is indication of selection of the cohort or subgroups for analysis and reporting on basis of the results (e.g. estimates not shown for all analyses).  *or*  There is no a-priori analysis plan or protocol.  *and*  There appears to be no issues with the exposure, multiple analyses (e.g., effect estimates were similar when different multiple analyses were used), or the selection or definition of subgroups, **but** there are inconsistencies/or no information between intended and reported analyses.  High risk of bias:  There is a high risk of selective reporting from multiple exposure measurements, outcomes measurements, or multiple analyses of data.  *or*  The cohort or subgroup is selected from a larger study for analysis and appears to be reported based on the results. (up to 2)  Very high risk of bias:  There is a high risk of selective reporting from multiple exposure measurements, *or* outcomes measurements, *or* multiple analyses of data *or* the cohort or subgroup is selected from a larger study for analysis and appears to be reported based on the results. (more than 2) |
| **Overall judgement** | Low risk of bias | The study is judged to be at low risk of bias for all domains. |
|  | Some concerns | The study is judged to be at low risk of bias or some concerns for all domains. |
|  | High risk of bias | The study is judged to be at high risk of bias in at least one domain, but no domains are at very high risk of bias. |
|  | Very high risk of bias | The study is judged to be at very high risk of bias in at least one domain. |

CVD cardiovascular disease; DHQ diet history questionnaire; FFQ food frequency questionnaire; HPFS Health Professional Follow up Study; NHS Nurses’ Health Study; SES socioeconomic status

^a^Triage: A (very) high risk of bias in the first domain leads to a triage. The further domains will not be considered for evaluation, as the overall judgement will already be at high risk of bias and will not be further influenced by any other domains (55)

# Supplemental References

Additional References

55. Sterne J, Reeves B, Savovic J, Higgins J. Assessing risk of bias in non-randomized studies of interventions: introduction to the ROBINS-S tool. ROBINS-I London Workshop October 2023. <www.riskofbias.info>: University of Bristol; 2023. p. <https://drive.google.com/file/d/1FyRqs88FTp42b6fQsT-5CeDif07Mn776/view>.

Included Studies

Uncategorized References

1. Chen G-C, Chai JC, Xing J, Moon J-Y, Shan Z, Yu B, et al. Healthful eating patterns, serum metabolite profile and risk of diabetes in a population-based prospective study of US Hispanics/Latinos. Diabetologia. 2022;65(7):1133-44.

2. Koloverou E, Panagiotakos DB, Georgousopoulou EN, Grekas A, Christou A, Chatzigeorgiou M, et al. Dietary Patterns and 10-year (2002-2012) Incidence of Type 2 Diabetes: Results from the ATTICA Cohort Study. Rev Diabet Stud. 2016;13(4):246-56.

3. Sobiecki JG, Imamura F, Davis CR, Sharp SJ, Koulman A, Hodgson JM, et al. A nutritional biomarker score of the Mediterranean diet and incident type 2 diabetes: Integrated analysis of data from the MedLey randomised controlled trial and the EPIC-InterAct case-cohort study. PLoS Med 2023;20(4):e1004221.

4. Vassou C, Yannakoulia M, Georgousopoulou EN, Chrysohoou C, Pitsavos C, Cropley M, et al. Irrational Beliefs, Dietary Habits and 10-Year Incidence of Type 2 Diabetes; the ATTICA Epidemiological Study (2002-2012). Rev Diabet Stud. 2021;17(1):38-49.

5. Wang DD, Qi Q, Wang Z, Usyk M, Sotres-Alvarez D, Mattei J, et al. The Gut Microbiome Modifies the Association Between a Mediterranean Diet and Diabetes in USA Hispanic/ Latino Population. J Clin Endocrinol Metab. 2022;107(3):e924-e34.

6. Cea-Soriano L, Pulido J, Franch-Nadal J, Santos JM, Mata-Cases M, Díez-Espino J, et al. Mediterranean diet and diabetes risk in a cohort study of individuals with prediabetes: propensity score analyses. Diabet Med. 2022;39(6):e14768.

7. Filippatos TD, Panagiotakos DB, Georgousopoulou EN, Pitaraki E, Kouli GM, Chrysohoou C, et al. Mediterranean Diet and 10-year (2002-2012) Incidence of Diabetes and Cardiovascular Disease in Participants with Prediabetes: The ATTICA study. Rev Diabet Stud. 2016;13(4):226-35.

8. Lago-Sampedro A, Oualla-Bachiri W, García-Serrano S, Maldonado-Araque C, Valdés S, Doulatram-Gamgaram V, et al. Protective Effect of High Adherence to Mediterranean Diet on the Risk of Incident Type-2 Diabetes in Subjects with MAFLD: The Di@bet.es Study. Nutrients [Internet]. 2024; 16(21).

9. Mirmiran P, Hosseini S, Bahadoran Z, Azizi F. Dietary pattern scores in relation to pre-diabetes regression to normal glycemia or progression to type 2 diabetes: a 9-year follow-up. BMC Endocr Disord. 2023;23(1):20.

10. Pérez-Ferre N, Del Valle L, Torrejón MJ, Barca I, Calvo MI, Matía P, et al. Diabetes mellitus and abnormal glucose tolerance development after gestational diabetes: A three-year, prospective, randomized, clinical-based, Mediterranean lifestyle interventional study with parallel groups. Clin Nutr. 2015;34(4):579-85.

11. 侯沂錚, 曾湘彤, 陳正裕, 吳晶惠. 地中海型飲食遵從度與健康成人罹患糖尿病前期之風險：病歷回溯研究. 台灣營養學會雜誌. 2024;48(1):1-12.

12. Wang Q, Schmidt AF, Lennon LT, Papacosta O, Whincup PH, Wannamethee SG. Prospective associations between diet quality, dietary components, and risk of cardiometabolic multimorbidity in older British men. Eur J Nutr. 2023;62(7):2793-804.

13. Wawro N, Pestoni G, Riedl A, Breuninger TA, Peters A, Rathmann W, et al. Association of Dietary Patterns and Type-2 Diabetes Mellitus in Metabolically Homogeneous Subgroups in the KORA FF4 Study. Nutrients [Internet]. 2020; 12(6).

14. Li Q, Wang X, Lu R, Zheng J, Chen J. Evaluation of Nutritional Interventions in Preventing Type 2 Diabetes Mellitus: A Randomized Controlled Trial. Curr Top Nutraceutical Res. 2024;22:424-30.

15. André P, Proctor G, Driollet B, Garcia-Esquinas E, Lopez-Garcia E, Gomez-Cabrero D, et al. The role of overweight in the association between the Mediterranean diet and the risk of type 2 diabetes mellitus: a mediation analysis among 21 585 UK biobank participants. Int J Epidemiol. 2020;49(5):1582-90.

16. Maroto-Rodriguez J, Ortolá R, Carballo-Casla A, Iriarte-Campo V, Salinero-Fort MÁ, Rodríguez-Artalejo F, et al. Association between a mediterranean lifestyle and Type 2 diabetes incidence: a prospective UK biobank study. Cardiovasc Diabetol. 2023;22(1):271.

17. Fan C, Wang W, Wang S, Zhou W, Ling L. Multiple dietary patterns and the association between long-term air pollution exposure with type 2 diabetes risk: Findings from UK Biobank cohort study. Ecotoxicol Environ Saf. 2024;275:116274.

18. Aryannezhad S, Imamura F, Mok A, Wareham NJ, Forouhi NG, Brage S. Combined associations of physical activity, diet quality and their trajectories with incidence of diabetes and cardiovascular diseases in the EPIC-Norfolk Study. Sci Rep. 2025;15(1):11261.

19. Wang Q, Schmidt AF, Wannamethee SG. Prospective Association of the Mediterranean Diet with the Onset of Cardiometabolic Multimorbidity in a UK-Based Cohort: The EPIC-Norfolk Study. J Nutr. 2024;154(12):3761-9.

20. Cespedes EM, Hu FB, Tinker L, Rosner B, Redline S, Garcia L, et al. Multiple Healthful Dietary Patterns and Type 2 Diabetes in the Women's Health Initiative. Am J Epidemiol. 2016;183(7):622-33.

21. Glenn AJ, Li J, Lo K, Jenkins DJA, Boucher BA, Hanley AJ, et al. The Portfolio Diet and Incident Type 2 Diabetes: Findings From the Women's Health Initiative Prospective Cohort Study. Diabetes Care. 2023;46(1):28-37.

22. Kechagia I, Tsiampalis T, Damigou E, Barkas F, Anastasiou G, Kravvariti E, et al. Long-Term Adherence to the Mediterranean Diet Reduces 20-Year Diabetes Incidence: The ATTICA Cohort Study (2002–2022). Metabolites [Internet]. 2024; 14(4).

23. Koloverou E, Panagiotakos DB, Pitsavos C, Chrysohoou C, Georgousopoulou EN, Grekas A, et al. Adherence to Mediterranean diet and 10-year incidence (2002–2012) of diabetes: correlations with inflammatory and oxidative stress biomarkers in the ATTICA cohort study. Diabetes Metab Res Rev. 2016;32(1):73-81.

24. Damigou E, Anastasiou C, Chrysohoou C, Barkas F, Tsioufis C, Pitsavos C, et al. Prevented fractions of cardiovascular disease cases, by long-term adherence to the Mediterranean diet; the ATTICA study (2002-2022). Nutr Metab Cardiovasc Dis. 2025;35(5):103777.

25. Eguaras S, Bes-Rastrollo M, Ruiz-Canela M, Carlos S, de la Rosa P, Martínez-González MA. May the Mediterranean diet attenuate the risk of type 2 diabetes associated with obesity: the Seguimiento Universidad de Navarra (SUN) cohort. Br J Nutr. 2017;117(10):1478-85.

26. Ruiz-Estigarribia L, Martínez-González MA, Díaz-Gutiérrez J, Sayón-Orea C, Basterra-Gortari FJ, Bes-Rastrollo M. Lifestyle behavior and the risk of type 2 diabetes in the Seguimiento Universidad de Navarra (SUN) cohort. Nutr Metab Cardiovasc Dis. 2020;30(8):1355-64.

27. Khalili-Moghadam S, Mirmiran P, Bahadoran Z, Azizi F. The Mediterranean diet and risk of type 2 diabetes in Iranian population. Eur J Clin Nutr. 2019;73(1):72-8.

28. Ramezan M, Asghari G, Mirmiran P, Tahmasebinejad Z, Azizi F. Mediterranean dietary patterns and risk of type 2 diabetes in the Islamic Republic of Iran. East Mediterr Health J. 2019;25(12):896-904.

29. Esfandiar Z, Hosseini-Esfahani F, Mirmiran P, Azizi F. Diet quality indices and the risk of type 2 diabetes in the Tehran Lipid and Glucose Study. BMJ Open Diabetes Res Care. 2022;10(5).

30. Jannasch F, Nickel DV, Kuxhaus O, Schulze MB. Longitudinally changed diet quality scores and their association with type 2 diabetes mellitus and cardiovascular diseases in the EPIC-Potsdam study. Sci Rep. 2024;14(1):13907.

31. Galbete C, Kröger J, Jannasch F, Iqbal K, Schwingshackl L, Schwedhelm C, et al. Nordic diet, Mediterranean diet, and the risk of chronic diseases: the EPIC-Potsdam study. BMC Med. 2018;16(1):99.

32. Jacobs S, Boushey CJ, Franke AA, Shvetsov YB, Monroe KR, Haiman CA, et al. A priori-defined diet quality indices, biomarkers and risk for type 2 diabetes in five ethnic groups: the Multiethnic Cohort. Br J Nutr. 2017;118(4):312-20.

33. Jacobs S, Harmon BE, Boushey CJ, Morimoto Y, Wilkens LR, Le Marchand L, et al. A priori-defined diet quality indexes and risk of type 2 diabetes: the Multiethnic Cohort. Diabetologia. 2015;58(1):98-112.

34. Martínez-González MA, de la Fuente-Arrillaga C, Nunez-Cordoba JM, Basterra-Gortari FJ, Beunza JJ, Vazquez Z, et al. Adherence to Mediterranean diet and risk of developing diabetes: prospective cohort study. Bmj. 2008;336(7657):1348-51.

35. Martínez-González MA, Montero P, Ruiz-Canela M, Toledo E, Estruch R, Gómez-Gracia E, et al. Yearly attained adherence to Mediterranean diet and incidence of diabetes in a large randomized trial. Cardiovasc Diabetol. 2023;22(1):262.

36. Salas-Salvadó J, Bulló M, Estruch R, Ros E, Covas MI, Ibarrola-Jurado N, et al. Prevention of diabetes with Mediterranean diets: a subgroup analysis of a randomized trial. Ann Intern Med. 2014;160(1):1-10.

37. Freisling H, Viallon V, Lennon H, Bagnardi V, Ricci C, Butterworth AS, et al. Lifestyle factors and risk of multimorbidity of cancer and cardiometabolic diseases: a multinational cohort study. BMC Medicine. 2020;18(1):5.

38. Romaguera D, Guevara M, Norat T, Langenberg C, Forouhi NG, Sharp S, et al. Mediterranean diet and type 2 diabetes risk in the European Prospective Investigation into Cancer and Nutrition (EPIC) study: the InterAct project. Diabetes Care. 2011;34(9):1913-8.

39. Abiemo EE, Alonso A, Nettleton JA, Steffen LM, Bertoni AG, Jain A, et al. Relationships of the Mediterranean dietary pattern with insulin resistance and diabetes incidence in the Multi-Ethnic Study of Atherosclerosis (MESA). Br J Nutr. 2013;109(8):1490-7.

40. Ahmad S, Demler OV, Sun Q, Moorthy MV, Li C, Lee IM, et al. Association of the Mediterranean Diet With Onset of Diabetes in the Women's Health Study. JAMA Netw Open. 2020;3(11):e2025466.

41. Bantle AE, Chow LS, Steffen LM, Wang Q, Hughes J, Durant NH, et al. Association of Mediterranean diet and cardiorespiratory fitness with the development of pre-diabetes and diabetes: the Coronary Artery Risk Development in Young Adults (CARDIA) study. BMJ Open Diabetes Res Care. 2016;4(1):e000229.

42. Chen GC, Koh WP, Neelakantan N, Yuan JM, Qin LQ, van Dam RM. Diet Quality Indices and Risk of Type 2 Diabetes Mellitus: The Singapore Chinese Health Study. Am J Epidemiol. 2018;187(12):2651-61.

43. Crawford B, Steck SE, Sandler DP, Merchant AT, Woo JMP, Park YM. Dietary patterns, socioeconomic disparities, and risk of type 2 diabetes in the Sister Study. Diabetes Res Clin Pract. 2023;204:110906.

44. de Koning L, Chiuve SE, Fung TT, Willett WC, Rimm EB, Hu FB. Diet-quality scores and the risk of type 2 diabetes in men. Diabetes Care. 2011;34(5):1150-6.

45. de León AC, Coello SD, González DA, Díaz BB, Rodríguez JC, Hernández AG, et al. Impaired fasting glucose, ancestry and waist-to-height ratio: main predictors of incident diagnosed diabetes in the Canary Islands. Diabet Med. 2012;29(3):399-403.

46. Hlaing-Hlaing H, Dolja-Gore X, Tavener M, James EL, Hodge AM, Hure AJ. Diet Quality and Incident Non-Communicable Disease in the 1946-1951 Cohort of the Australian Longitudinal Study on Women's Health. Int J Environ Res Public Health. 2021;18(21).

47. Hodge AM, Karim MN, Hébert JR, Shivappa N, de Courten B. Association between Diet Quality Indices and Incidence of Type 2 Diabetes in the Melbourne Collaborative Cohort Study. Nutrients. 2021;13(11).

48. Lin R, Chien KL, Tsai MC, Wang YJ, Hsu LY. Association between a priori and a posteriori dietary patterns and the risk of type 2 diabetes: a representative cohort study in Taiwan. J Nutr Sci. 2023;12:e16.

49. O'Connor LE, Hu EA, Steffen LM, Selvin E, Rebholz CM. Adherence to a Mediterranean-style eating pattern and risk of diabetes in a U.S. prospective cohort study. Nutr Diabetes. 2020;10(1):8.

50. Rai SK, Gortmaker SL, Hu FB, Kanaya AM, Kandula NR, Sun Q, et al. A South Asian Mediterranean-style diet is associated with favorable adiposity measures and lower diabetes risk: The MASALA cohort. Obesity (Silver Spring). 2023;31(6):1697-706.

51. Rossi M, Turati F, Lagiou P, Trichopoulos D, Augustin LS, La Vecchia C, et al. Mediterranean diet and glycaemic load in relation to incidence of type 2 diabetes: results from the Greek cohort of the population-based European Prospective Investigation into Cancer and Nutrition (EPIC). Diabetologia. 2013;56(11):2405-13.

52. Tison SE, Shikany JM, Long DL, Carson AP, Cofield SS, Pearson KE, et al. Differences in the Association of Select Dietary Measures With Risk of Incident Type 2 Diabetes. Diabetes Care. 2022;45(11):2602-10.

53. Ying Z, Fu M, Fang Z, Ye X, Wang P, Lu J. Mediterranean diet lowers risk of new-onset diabetes: a nationwide cohort study in China. Nutr J. 2024;23(1):131.

54. Forouhi NG, Wareham NJ. The EPIC-InterAct Study: A Study of the Interplay between Genetic and Lifestyle Behavioral Factors on the Risk of Type 2 Diabetes in European Populations. Curr Nutr Rep. 2014;3(4):355-63.
